# The core bacterial microbiome of banana (*Musa* spp.)

**DOI:** 10.1186/s40793-022-00442-0

**Published:** 2022-09-08

**Authors:** Henry W. G. Birt, Anthony B. Pattison, Adam Skarshewski, Jeff Daniells, Anil Raghavendra, Paul G. Dennis

**Affiliations:** 1 School of Earth and Environmental Sciences, The University of Queensland, Brisbane, QLD 4072 Australia; 2 Department of Agriculture and Fisheries, Centre for Wet Tropics Agriculture, 24 Experimental Station Road, South Johnstone, QLD 4859 Australia

**Keywords:** Fusarium, Plant growth promotion, Plant disease, Biocontrol, Plant protection, Sustainable agriculture

## Abstract

**Background:**

Bananas (*Musa* spp.) are a globally significant crop and are severely afflicted by diseases for which there are no effective chemical controls. Banana microbiomes may provide novel solutions to these constraints but are difficult to manage due to their high diversity and variability between locations. Hence ‘common core’ taxa, which are a subset of the microbiome that frequent all, or most, individuals of a host species, represent logical targets for the development of microbiome management approaches. Here, we first performed a pot experiment to characterise the effects of two factors that are likely to differ between farms (viz. edaphic conditions and host genotype) on bacterial diversity in bulk soil and seven plant compartments. From this experiment, we created shortlisted core ‘candidates’ that were then refined using a survey of 52 field-grown *Musa* spp. We confirmed the importance of the core through network analysis and by comparing the sequences of our core taxa with those reported in 22 previous studies.

**Results:**

Diversity was found to differ between plant compartments and soils, but not genotypes. Therefore, we identified populations that were frequent across most plants irrespective of the soil in which they were grown. This led to the selection of 36 ‘common core’ bacteria, that represented 65–95% of the dominant taxa in field-grown plants and were identified as highly interconnected ‘hubs’ using network analysis – a characteristic shown to be indicative of microbes that influence host fitness in studies of other plants. Lastly, we demonstrated that the core taxa are closely related to banana-associated bacteria observed on five other continents.

**Conclusions:**

Our study provides a robust list of common core bacterial taxa for *Musa* spp. Further research may now focus on how changes in the frequencies and activities of these most persistent taxa influence host fitness. Notably, for several of our core taxa, highly similar populations have already been isolated in previous studies and may be amenable to such experimentation. This contribution should help to accelerate the development of effective *Musa* spp. microbiome management practices.

**Supplementary Information:**

The online version contains supplementary material available at 10.1186/s40793-022-00442-0.

## Background

Bananas (*Musa* spp.) are among the world’s most-produced crops and represent an important food staple in many countries [1]. Abiotic and biotic stresses undermine banana production and include diseases that can persist in soils for long periods, and for which there are no effective chemical controls [2, 3]. Like other macro-organisms, *Musa* spp. host diverse microbial communities that influence their health and nutrition. Management of these communities may offer novel solutions to production constraints but is challenging due to the complexity of plant–microbe interactions and their dependency on environmental conditions. Hence, an intervention that works on one farm may be ineffective on another due to differences in the microbiome. Despite this variability, evidence indicates that all plants harbour a subset of ‘core taxa’ with which they are persistently associated across diverse environmental gradients [4–6]. The pervasive nature of these organisms, suggests that they play important roles in the regulation of host function [7–9], and presents an opportunity to focus research efforts on a relatively small, impactful and frequently encountered subset of taxa. This approach may increase the likelihood of microbiome management interventions being effective on farms with different abiotic and biotic characteristics.

To identify a ‘core bacterial microbiome’ of banana, viz. a shortlist of bacterial taxa that are shared by all or most *Musa* spp., it is necessary to characterise the microbiome in as many settings as possible. While Cavendish cultivars dominate commercial banana production, many genotypes are grown around the world, on land with differing edaphic conditions [10]. Hence, the extent to which these factors influence *Musa* spp. microbiomes must be assessed and considered appropriately when designating core taxa. For *Musa* spp., the effects of edaphic conditions and genotype on bacterial diversity are poorly understood. Nonetheless, results from a wide-range of other plant species indicate large differences between soils, and significant but much smaller effects of genotype that are positively associated with host phylogenetic distance [6].

Another important host-associated factor to consider when defining core taxa, is that microbial communities differ between plant compartments [11]. In general, the composition of root microbiomes is most strongly determined by edaphic conditions, as soil is the primary source of root-colonising microbes [12, 13]. In contrast, leaf microbiomes, are distinct from belowground microbial communities [14, 15] and comprise a larger proportion of taxa derived from aerial sources [16]. Understanding these spatial patterns is likely to be important for the design of effective microbiome management approaches. For example, *Fusarium oxysporum* f. sp. *cubense* (*Foc*), the causal agent of Fusarium wilt of banana (FWB), tends to infect at root apices, and then migrate to the leaves via the xylem, rhizome, and outer leaf sheaths of the pseudostem [17]. Hence, determining the core taxa associated with these compartments may help to identify where relevant *Foc* suppressive populations occur and contribute to the development of biological control strategies.

Here, we define the common core bacterial microbiome of *Musa* spp. This process began with pot experiments aiming to: (1) characterise the bacterial communities associated with different *Musa* spp. plant compartments; (2) determine whether *Musa* spp. microbiomes vary with edaphic conditions and between host genotypes; and (3) identify a shortlist of ‘candidate core’ bacterial taxa that are shared by all *Musa* spp. irrespective of differences in edaphic conditions and host genotype. We then performed a survey of 52 distinct field-grown *Musa* spp., comprising a further 700 samples. The aims of our field experiment were to determine whether the ‘candidate core’ taxa identified in our pot experiment were: (1) important in mature field-grown plants; and (2) present in a much larger range of genotypes. Finally, we compared the sequences of our refined core bacterial taxa with the SILVA database, and data from 22 previous studies of *Musa* spp.-associated bacteria. This meta-analysis demonstrated that our core taxa are closely related to bacterial populations that have previously been observed in association with *Musa* spp. worldwide.

## Methods

### Pot experiments

*Experimental setup* Soil was collected (0–30 cm depth) from five *Musa* (AAA Group, Cavendish Subgroup) ‘Williams’ production sites in The Wet Tropics of North Queensland – the primary banana-producing area of Australia (Additional file 1: Table S1). Within this region, soils are grouped into series, which have horizons derived from similar parent material, and with similar properties and arrangement in the profile [18–20]. Each soil in our study represents one of the dominant soil series of the region (Innisfail, In; Liverpool, Li; Pin Gin, Pg; Tully, Tu; and Tolga, To), and has distinct edaphic properties (Additional file 1: Table S1). All soils were sieved to < 8 mm and then placed in 15 cm diameter pots, with 1 kg of soil (fresh weight) per pot. Ten pots of each soil were directly planted with sterile ‘Williams’ tissue culture plantlets (Kool Bananas tissue culture facility, Mission Beach). These plants were used to assess the impact of edaphic properties on the bacterial microbiome of *Musa* spp. An additional 20 pots were then filled with Innisfail soil: 10 for *Musa* (AAB Group, Pome Subgroup) ‘Lady Finger’, and 10 for *Musa* (AAAB Group, Prata Anã x SH-3142) 'Goldfinger' plantlets (Department of Agriculture and Fisheries, Nambour tissue culture facility). Together with the 10 pots of ‘Williams’ in Innisfail soil, these plants were analysed as a separate experiment to assess the impact of host genotype on the bacterial microbiome of *Musa* spp. ‘Williams’ and ‘Lady Finger’ represent c. 97% and 3% of banana production in Australia, respectively [21]. ‘Goldfinger’ is not widely grown but has a tetraploid hybrid genome and is more resistant to Fusarium Wilt [22]. All plants were grown in a glasshouse with an ambient air temperature of 22–31 °C and watered with a fixed sprinkling system twice daily for 5 min to maintain relative humidity greater than 60%. Trays were placed at the base of the pots to avoid water loss and the treatments were arranged in a randomised design. All plants were harvested after three months of growth.

*Sample collection* For each plant we collected samples from eight compartments: (1) bulk soil (BS), (2) apical ectorhizosphere (AER), (3) basal ectorhizosphere (BER), (4) apical endorhizosphere (AEnR), (5) basal endorhizosphere (BEnR), (6) rhizome (R; also known as the corm), (7) pseudostem (PS), and (8) leaves (L), as previously described [23]. This design yielded 560 samples (70 plants × 8 compartments). Briefly, the first three most recently emerged leaves were removed and finely chopped to form the leaf samples. Plants were then removed from pots and shaken to separate and collect bulk soil samples. Roots were cut 50 mm from the base and apex, placed in separate sterile 50 ml falcon tubes, and retained at 4 °C in a refrigerator for later processing on the same day. After washing in deionised water, the rhizome and pseudostem were separated with a knife. Rhizome samples were surface sterilised in 4% NaOCl for 5 min and washed three times in sterile deionised water [24]. All samples were then stored at − 20 °C after collection. Tubes containing roots were removed from the refrigerator, filled with 40 ml sterile 1X phosphate buffer saline (PBS) solution, and then vortexed for 1 min, sonicated for 1 min, and vortexed for another 1 min. Basal and apical roots were then transferred to fresh tubes using sterile forceps, and the resulting slurries were centrifuged for 15 min at 27 RCF. The clear supernatants were removed, and the soil pellets were retained the basal and apical ectorhizosphere samples. Having removed the ectorhizosphere soil, basal and apical roots were washed in sterile distilled water by repeatedly vortexing, then removing and replacing water until it appeared clear after vortexing (c. 5–8 times). Roots were then sonicated in 50 ml of water for 1 min to remove remaining rhizoplane organisms, surface sterilised in 4% NaOCl for 5 min, and then washed three times in sterilised distilled water. These sterile roots formed the basal and apical endorhizosphere samples. All samples were then stored at − 20 °C until further processing.

### Field experiment

*Experimental design and sample collection* Bulk soil, root, pseudostem, and leaf samples were collected from 55 mature field-grown plants, representing 52 genotypes from the Australian Banana Germplasm Collection (17.60667° S, 145.9983° E) which is maintained at the Centre for Wet Tropics Agriculture, South Johnstone, Queensland (Additional file 1: Table S2). These genotypes included dessert bananas, cooking bananas, and wild *Musa* spp. (Additional file 1: Table S2). Obvious symptoms of pest or pathogen pressure were not apparent upon visual inspection. While the soil at the site is of Innisfail series, it was from a different location to that used in the pot experiment. For each plant we collected samples in triplicate. Bulk soil and a mixture of apical and basal roots were collected from the base of three pseudostems at different stages of flowering (emergent, immature, and mature). A sample of each pseudostem was then collected using a sterile corer, and the central section of the second most recently emerged leaf was collected with sterile cutting tools. All samples were stored on ice in the field, and then transferred to − 20 °C in the lab, except for root samples. Roots were processed as described above to obtain ectorhizosphere and endorhizosphere samples, which were then frozen at − 20 °C.

### DNA extraction, and PCR and sequencing of 16S rDNA

*DNA extraction* Samples were transferred from frozen storage to a freeze drier, lyophilised, and then ground using a TissueLyser II (Qiagen). For each sample, DNA was extracted from 150 mg of fine powder using DNeasy PowerSoil HTP kits (Qiagen) according to the manufacturer’s instructions, except for an extra 400 µl of PowerBead solution, which ensured sufficient moisture within each well.

*PCR* Universal bacterial 16S rRNA genes were amplified by polymerase chain reaction (PCR) using the primers 799F (5’- AAC MGG ATT AGA TAC CCK G-3’) and 1193R (5’- ACG TCA TCC CCA CCT TCC-3’), each modified on the 5’ end to contain the Illumina overhang adapter for compatibility with the P5 and i7 Nextera XT indices, respectively. PCR reactions contained: 2 µl of DNA sample in 5X Phire Green Reaction Buffer (Thermo Fisher), 100 µM of each of the dNTPs (Invitrogen), 0.4 µl of Phire Green Hot Start II DNA Polymerase (Thermo Fisher), and 10 mM of each primer. This reaction was made up to a total volume of 20 µl with molecular biology grade water. Thermocycling conditions were as follows: 98 °C for 45 s, then 35 cycles of 98 °C for 5 s, 56 °C for 5 s, 72 °C for 6 s, and 72 °C for 1 min. Amplifications were performed using a SimpliAmp^®^ 96-well Thermocycler (Applied Biosystems). Blank DNA extraction controls, and no-template PCR controls were verified to be negative using gel electrophoresis.

Sequencing Amplicons were purified using magnetic beads [25] and subjected to dual indexing using the Nextera XT Index Kit (Illumina) according to the manufacturer’s instructions. Indexed amplicons were purified using magnetic beads and then quantified using a PicoGreen dsDNA Quantification Kit (Invitrogen). Equal concentrations of each sample were pooled and sequenced on an Illumina MiSeq using 30% PhiX Control v3 (Illumina) and a MiSeq Reagent Kit v3 (600 cycles; Illumina) according to the manufacturer’s instructions.

### Processing of sequence data

By combining all pot and field data in a single bioinformatics analysis, it was possible to investigate the same OTUs in both settings. As the candidate-core OTUs were identified using the pot experiment only, this approach enabled us to use the field survey as an independent dataset for validation purposes. Sequence data were processed using a modified UPARSE workflow [26]. Briefly, demultiplexing and primer removal was performed using cutadapt in QIIME2 (v2017.9.0) [27]. Then in USEARCH (v10.0.240) [28], fastx_truncate was used to trim (250 bp) forward reads, which were quality filtered using fastq_filter (− fastq_maxee = 1.0), and then mapped against representative sequences, generated using fastx_uniques and cluster_otus (sequence similarity = 0.97), to create an operational taxonomic unit (OTU) table using otutab. OTUs were assigned SILVA 138 [29] taxonomy using BLASTN (v2.3.0 +) [30] in QIIME2, and those classified as chloroplasts, mitochondria, archaea or eukaryotes were removed from the OTU table using BIOM [31]. Representative bacterial sequences were aligned using MAFFT (v7.221) [32] and masked using QIIME2 to calculate phylogenetic distance and generate a midpoint-rooted phylogenetic tree using FastTree (v2.1.9) [33]. Samples were rarefied to 1000 reads, and the mean numbers of observed (Sobs) and predicted (Chao1) [34] OTUs, as well as Shannon’s Diversity Index [35], and Faith's Phylogenetic Diversity Index (Faith's PD) [36] were calculated using QIIME2. Variation in the composition of microbial communities between samples was investigated from a taxonomic (Hellinger transformed relative OTU abundances) [37] and phylogenetic (weighted UniFrac distances) perspective [38].

### Data analyses for the pot experiments

*Effects of compartment and soil/genotype* The effects of soil and genotype were analysed as separate experiments – one focussing on a single genotype in five soils, and the other focussing on three genotypes in one soil. The main and interactive effects of compartment (bulk soil, and plant compartments) and soil/genotype on bacterial alpha diversity (Sobs, Chao1, Shannon, and Faith’s PD) were assessed using analysis of variance (ANOVA) with Tukey’s HSD post hoc analyses as implemented using the R (v4.0.3) [39] packages *base* and *agricolae* [40]. The main and interactive effects of compartment and soil/genotype on the composition of bacterial communities (*i.e*. beta diversity as represented by Hellinger transformed OTU relative abundances and weighted UniFrac distances) were assessed using Permutational Multivariate Analysis of Variance (PERMANOVA) [41] as implemented in *vegan* [42]. The extent to which the microbiomes associated with different compartments resembled one another was visualised using detrended correspondence analysis (DCA). In addition we used SourceTracker, a Bayesian approach implemented through QIIME [43], to determine the proportion of shared taxa between compartments. In this analysis, each compartment was analysed as a source and compared to all others as a sink.

*Defining ‘candidate core’ bacterial taxa* For each level of a significant factor (*i.e.* soil or genotype), we selected candidate-core OTUs on the basis that they were in ≥ 50% of replicates within one or more compartments, at a mean relative abundance of ≥ 0.5% where present.

### Data analyses for the field survey

*Assessing the importance of candidate core taxa using networks* The importance (*i.e*. centrality) of candidate-core relative to non-core OTUs in community interactions were first assessed using network analysis. A network summarising community interactions for the field dataset was inferred using the R package *SpiecEasi* [44]*.* The network was inferred using OTUs with > 15% prevalence, and at least one occurrence where its relative abundance was ≥ 1% to reduce computational load and spurious connections. Network centrality metrics were calculated for each node (OTU) using the *igraph* and *centiserve* R packages [45, 46] and included: (1) degree, (2) weighted degree, (3) betweenness centrality, (4) closeness centrality, (5) Markov centrality, and (6) PageRank score. Wilcoxon rank-sum tests were used to assess whether these ‘importance’ metrics differed significantly between ‘candidate core’ and non-core OTUs using the *base* R package. Gephi [47] was used for network projection and visualisation.

*Assessing the importance of candidate core taxa based on their abundance and prevalence in field-grown plants* Next, we determined whether the candidate-core OTUs were found within the ‘key constituents’ of field-grown plants, which we defined as OTUs that were present in at least one plant compartment, in ≥ 50% of plants, at ≥ 0.5% relative abundance. Candidate core OTUs that were listed among the ‘key constituents’ of field-grown plants were elevated to full core status, while those that were found only in potted plants were dismissed.

### Comparing core OTUs with sequences from 22 previous studies

Finally, we sought to evaluate whether close relatives of our core OTUs have been observed in previous studies of *Musa* spp. bacterial communities. Accordingly, we downloaded bacterial 16S rRNA gene amplicon sequences from 22 previous studies covering five continents (Additional file 1: Table S3; Fig. S1). All Sanger datasets had been quality filtered prior to being made publicly available; therefore, no further processing was necessary. For Illumina and 454 datasets, primer and barcode sequences were removed in QIIME v1.9.1 [48] using multiple_extract_barcodes.py. Each 454 dataset was homopolymer-error corrected using Acacia version 1.52 [49]. Illumina and 454 datasets were then quality filtered (qual score = 25) using multiple_split_libraries_fastq.py in QIIME v1.9.1.

These data were generated using different sequencing platforms (Sanger, 454, and Illumina) and by amplifying different regions of 16S rRNA genes, hence, direct blast comparisons with our core OTUs were not possible. For this reason, our core OTU sequences were first queried against full length sequences in SILVA 138 using BLASTN (v2.3.0 +). The full-length sequences with the best matches to our core taxa were then extracted and, for each core taxa, converted into a blast database. The quality filtered sequences from previous studies were then queried against each core taxa blast database. The highest percentage sequence similarity from each study to each core taxa blast database was then reported for all hits over > 95% of the query sequence length.

## Results

### Effects of compartment and soil/genotype

**Fig. 1:**
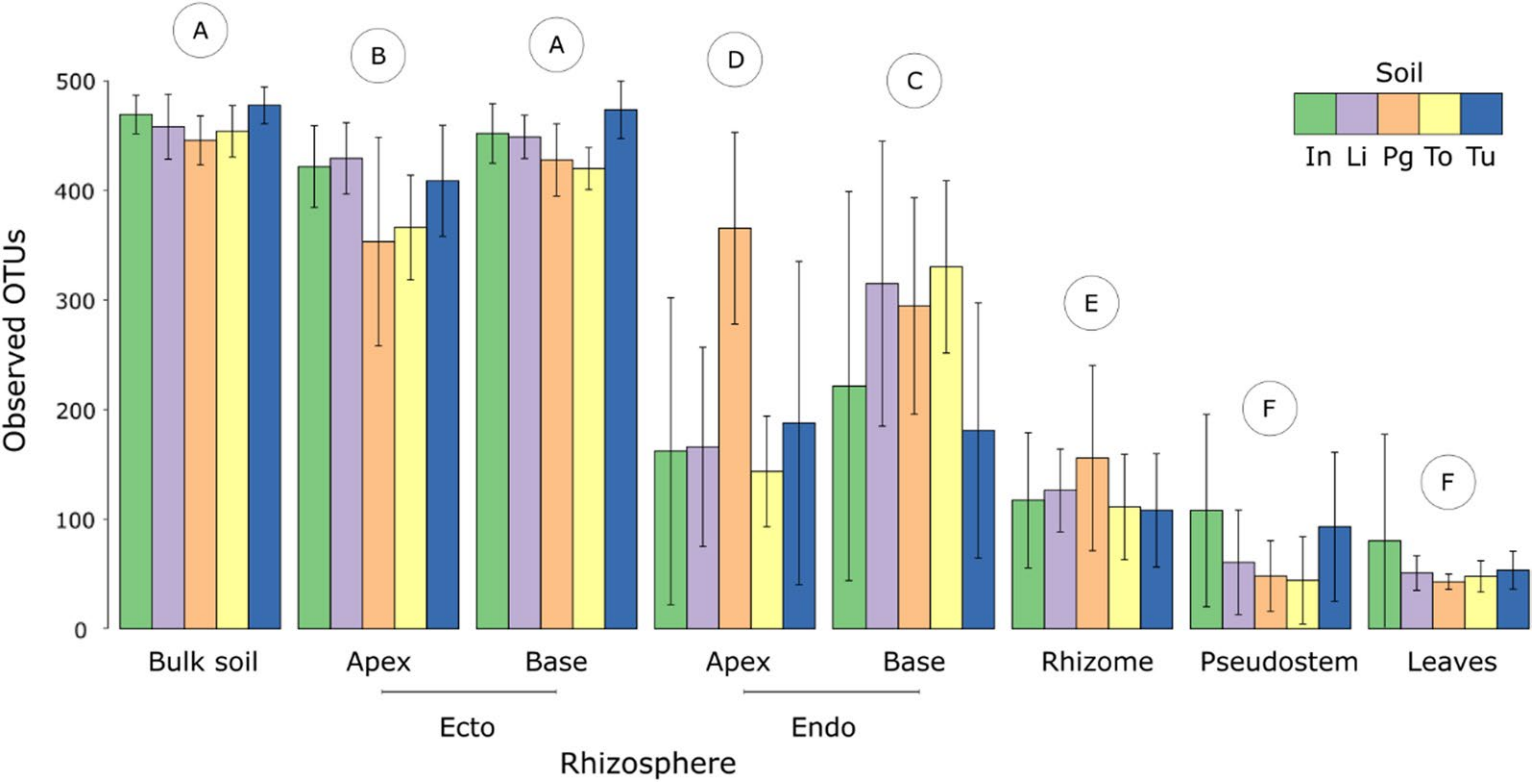
Numbers of observed bacterial OTUs associated with different plant compartments of *Musa* (AAA Group, Cavendish Subgroup) ‘Williams’ grown in pots with five distinct soils. Error bars represent standard errors of the means. The letters in circles indicate compartments that differ across soils according to Tukey post hoc tests. Members of the same groupings share the same letter. Abbreviations for soils are as follows: In – Innisfail, Li – Liverpool, Pg – Pin Gin, To – Tolga, and Tu – Tully

**Fig. 2:**
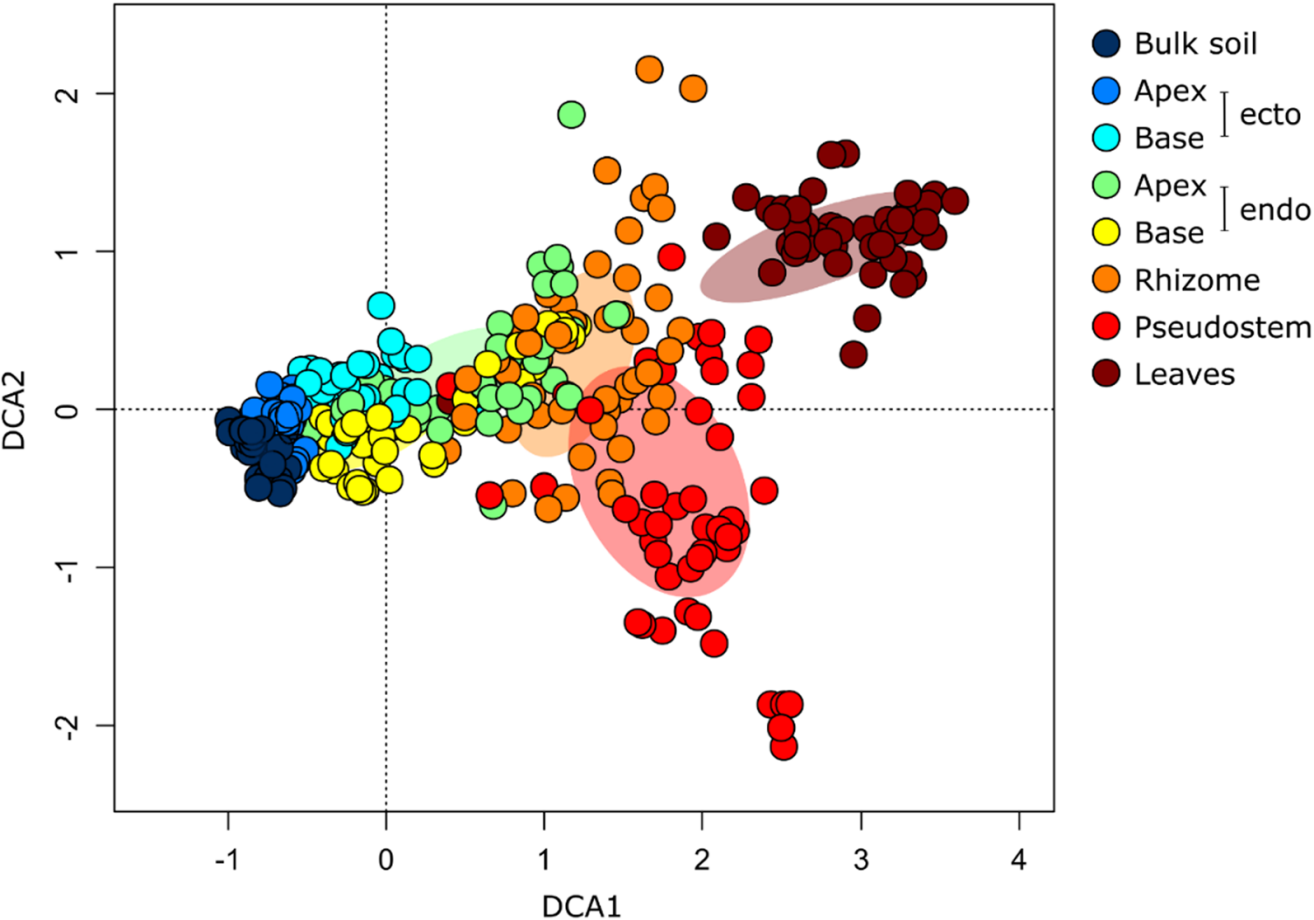
Detrended correspondence analysis (DCA) ordination highlighting differences in the composition of bacterial communities (Hellinger transformed OTUs) associated with the different plant compartments of *Musa* (AAA Group, Cavendish Subgroup) ‘Williams’. Each compartment is represented by 50 replicates comprising 10 samples from plant grown in five distinct soils. The ellipses represent standard deviations of the group centroids. Abbreviations are as follows: endo – endorhizosphere, ecto – ectorhizosphere

*Compartment* Bacterial diversity and community composition differed significantly between compartments, and this effect was considerably stronger than those associated with soil or host-genotype (Tables 1, Additional file 1: Table S4, S5). The alpha diversity of bulk soil and ectorhizosphere communities was more extensive than that of endophytic communities and declined towards the leaves (Fig. 1). The composition of bacterial communities was more similar in more proximal compartments, with bulk soil and ectorhizosphere communities being most dissimilar to those associated with the pseudostem and leaves (Fig. 2). This finding was also supported by SourceTracker [43] – a tool used to generate Bayesian estimates of the proportions of each community derived from other compartments (Additional file 1: Table S6). For example, this analysis indicated that 96% of OTUs associated with leaves were derived from the pseudostem, while only 7% were derived from bulk soil (Additional file 1: Table S6).

**Table 1:** The impacts of soil, genotype, and plant compartment on observed bacterial OTUs and community composition (Hellinger transformed OTUs) using ANOVA and PERMANOVA, respectively

Predictor variable	Number of observed OTUs			Community composition		
	d.f.	*F* value	*P* value	*F* value	*R*^2^ (%)	*P* value
Compartment	7	268.6	< 0.001***	26.1	29.3	< 0.001***
Soil	4	1.6	0.167	6.2	4.0	< 0.001***
Compartment: Soil	28	4.1	< 0.001***	2.2	10.0	< 0.001***
Compartment	7	113.2	< 0.001***	16.2	33.3	< 0.001***
Genotype	2	0.6	0.569	1.3	0.8	0.110
Compartment: Genotype	14	0.6	0.845	1.1	4.6	0.115

*P* < 0.05*, *P* < 0.01**, *P* < 0.001***

These results derive from our pot experiment which included five distinct soils, three *Musa* spp. genotypes, and eight compartments, each with 10 replicates

**Fig. 3:**
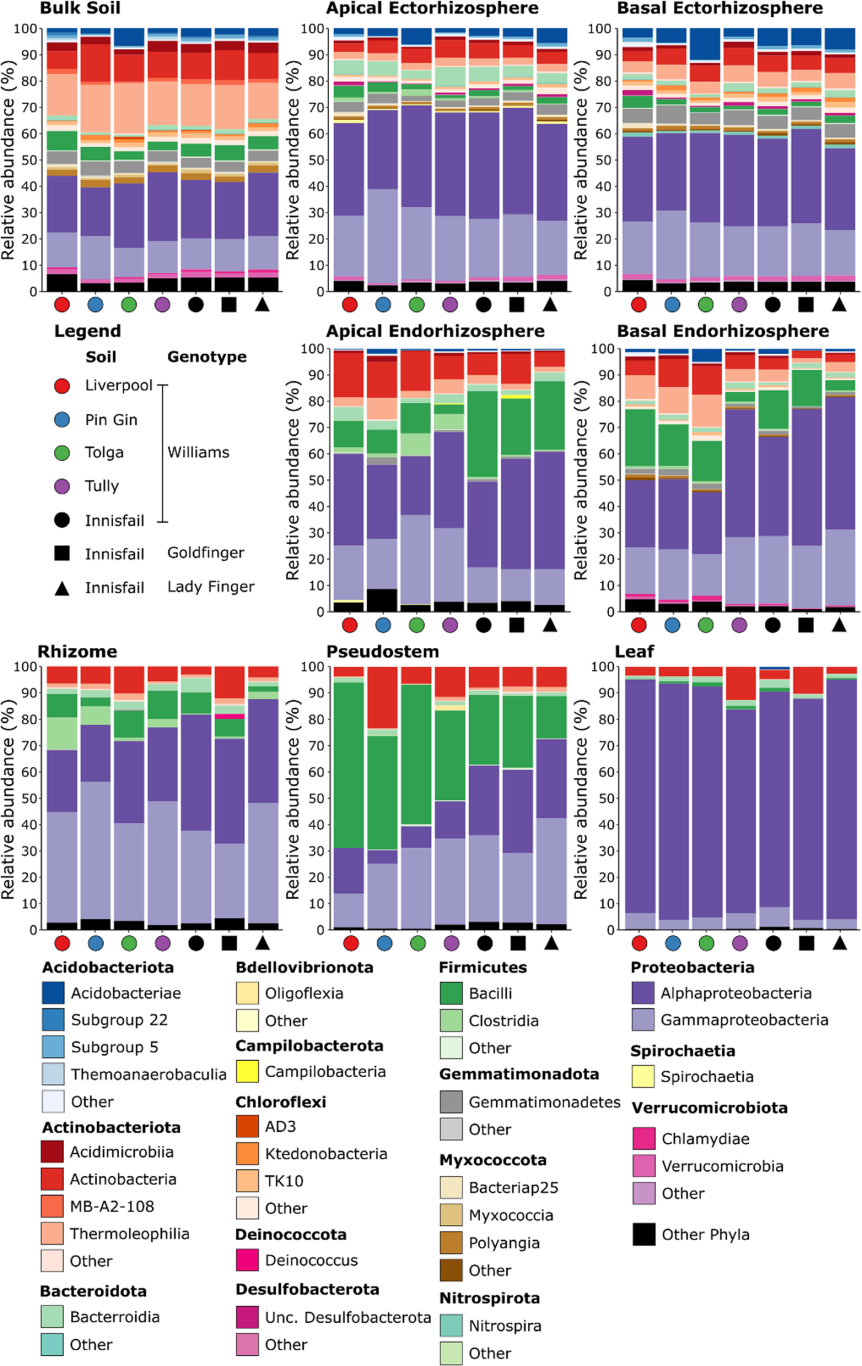
The mean relative frequencies of bacterial classes in different plant compartments associated with *Musa* (AAA Group, Cavendish Subgroup) ‘Williams’ grown in pots with five distinct soils, and two other *Musa* spp. genotypes grown in pots containing an Innisfail series soil. Within each phylum, classes represented at < 1% mean relative abundance are grouped as other

*Musa* spp. bacterial communities were dominated by representatives of the Acidobacteriota, Actinobacteriota, Bacteroidota, Firmicutes, Gemmatimonadota, Nitrospirota, Proteobacteria, and Verrucomicrobiota (Fig. 3). Acidobacteriota were frequent in soil and ectorhizosphere compartments, but not in endophytic compartments, which were strongly associated with members of the Bacilli (Fig. 3). Representatives of the actinobacteriotal classes Actinobacteria and Thermoleophilia were found throughout the plant, although members of the latter were not frequent on leaves (Fig. 3). By far the most frequent bacteria were members of the Alpha- and Gamma-proteobacteria (Fig. 3), which represented 14–85% and 6–43% mean relative abundance within each compartment, respectively (Fig. 3).

*Soil/genotype* In addition to compartment, bacterial diversity and community composition differed significantly between soils, but not host genotypes (Table 1 and Additional file 1: Table S4, S5). Between soils, differences in alpha diversity were generally apparent in belowground (bulk soil and rhizosphere) compartments but not elsewhere (Table 2 and Additional file 1: Table S7). Differences in bacterial community composition between soils were not detected in leaves but were apparent in all other compartments (Table 2 and Additional file 1: Table S8). In summary, our results demonstrate that the diversity and composition of bacterial communities associated with *Musa* spp. differs between the various compartments of a plant and the soil in which it is grown, but not between the genotypes assessed.

**Table 2:** The influence of soil on the numbers of observed bacterial OTUs and community composition (Hellinger transformed OTUs) within each compartment, as assessed by ANOVA and PERMANOVA, respectively

Compartment	Number of observed OTUs		Community composition		
	*F* value	*P* value	*F* value	*R*^2^ (%)	*P* value
Bulk soil	3.2	0.022*	3.8	25.2	< 0.001***
Apical ectorhizosphere	3.5	0.014*	3.1	21.8	< 0.001***
Apical endorhizosphere	7.0	< 0.001***	2.5	19.5	< 0.001***
Basal ectorhizosphere	6.9	< 0.001***	4.2	27.0	< 0.001***
Basal endorhizosphere	2.7	0.045*	2.5	18.3	< 0.001***
Rhizome	1.1	0.385	1.7	13.3	< 0.001***
Pseudostem	2.2	0.085	3.1	22.9	< 0.001***
Leaf	1.0	0.394	1.2	9.6	0.226

*P* < 0.05*, *P* < 0.01**, *P* < 0.001***

The results are for the *Musa* (AAA Group, Cavendish Subgroup) ‘Williams’ plants grown in five distinct soils in our pot experiment

### Defining ‘candidate core’ bacterial taxa

Based on our findings, we sought to define a core microbiome of banana comprising taxa found in all five soils within the plant compartments characterised. For each soil, we selected candidate-core OTUs on the basis that they were in at least five of ten replicates within one or more compartments, at a mean relative abundance of ≥ 0.5% where present (Fig. S2). This approach yielded a total of 228 candidate-core OTUs (Additional file 1: Table S9). Of these, 47 were shared between all soils within at least one compartment and hence maintained candidate-core status (Additional file 1: Figs. S3 and S4). The remainder lost candidate-core status and were mostly specific to a particular soil (Additional file 1: Fig. S2; Table S9).

### Confirming ‘core status’ in mature field-grown Musa spp. plants

*Differences between pot and field microbiomes* While our pot experiments enabled the effects of genotype and edaphic conditions to be studied in isolation of other potential drivers of bacterial diversity, we were conscious of the need to consider our results under more realistic conditions. Hence, we performed a survey of mature field-grown *Musa* spp. to facilitate comparisons of pot and field associated plant microbiomes. Field-grown *Musa* spp. microbiomes were dominated by representatives of the same phyla as pot plants (Fig. 3 and Additional file 1: Fig. S5). As for pot plants, compartment was a strong predictor of bacterial diversity in the field (Table 3). Nonetheless, depending on the compartment studied, there were relatively small but significant differences attributable to setting (field or pot; Table 3). Relative to pot plants, field microbiomes were less diverse in belowground compartments and more diverse in aboveground compartments (Additional file 1: Fig. S6). In terms of community composition, differences between settings were most pronounced in the bulk soil and ectorhizosphere (Additional file 1: Fig. S7).

**Table 3:** The impact of the setting *Musa* spp. were grown in (field vs. pot) and plant compartment on their associated observed bacterial OTUs and community composition (Hellinger transformed OTUs) as assessed using ANOVA and PERMANOVA, respectively

Predictor variable	Number of observed OTUs			Community composition (Hellinger distance)		
	d.f.	*F* value	*P* value	*F* value	*R*^2^ (%)	*P* value
Setting	1	5.3	0.022*	60.2	3.6	< 0.001***
Compartment	4	860.7	< 0.001***	85.2	20.5	< 0.001***
Setting: compartment	4	45.2	< 0.001***	22.5	5.2	< 0.001***

*P* < 0.05*, *P* < 0.01**, *P* < 0.001***

**Fig. 4:**
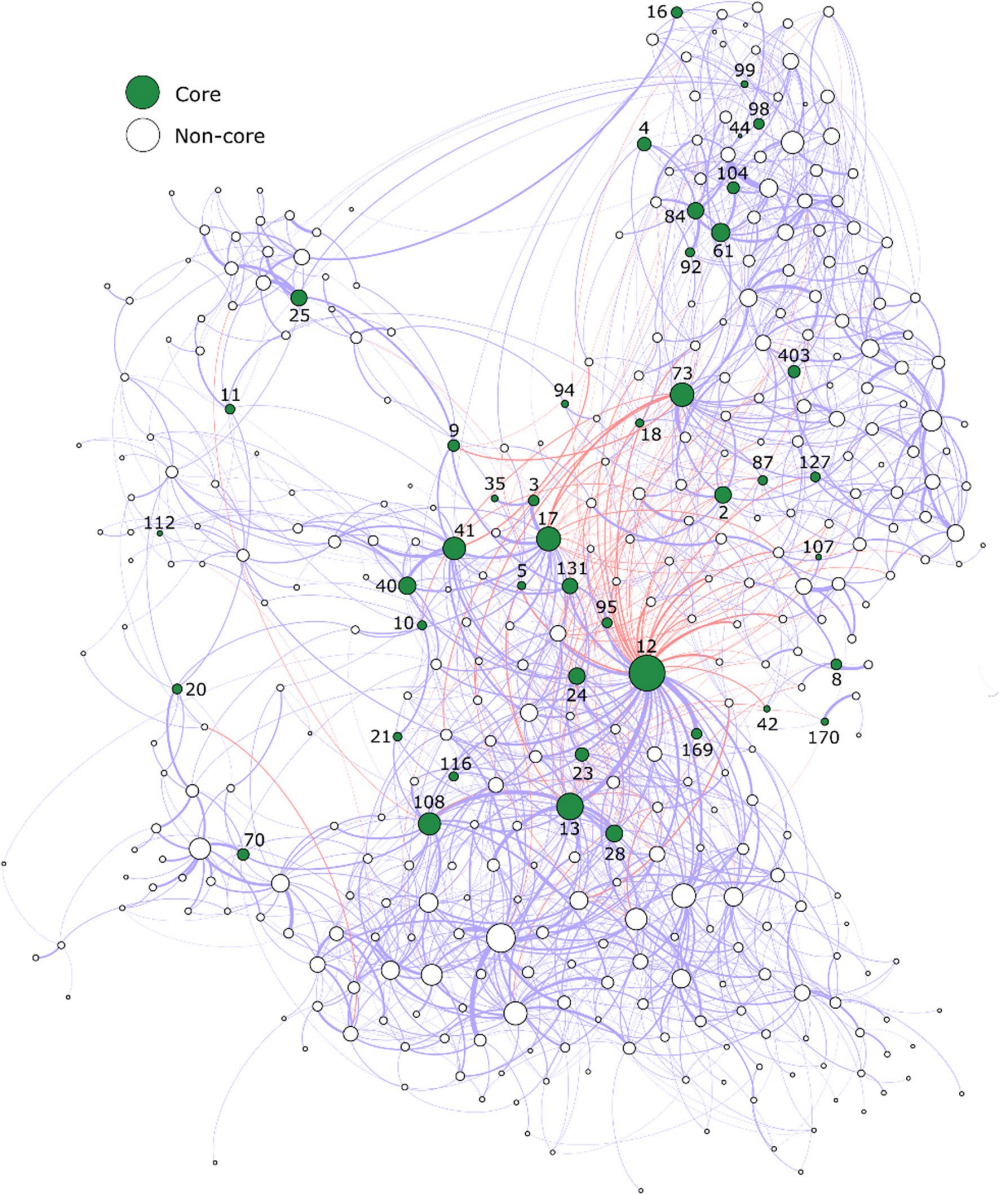
A SPIEC-EASI network graph showing the co-occurrences of core and non-core bacteria in field-grown *Musa* spp. Nodes are coloured by core status and size is positively associated with weighted degree. Edge colours represent positive (blue) and negative (red) associations between taxa. Edge width is positively associated with the coefficient for the co-occurrence between the taxa. The numbers next to the nodes represent OTU IDs

*Assessing the importance of candidate-core OTUs using networks* In light of our findings, we used network analysis to infer whether the candidate-core OTUs identified in our pot experiments were important members of field-grown *Musa* spp. microbiomes. Interactions between bacterial populations for the field dataset were inferred using a SPIEC-EASI network. Centrality metrics, representing the relative importance of each population, were then calculated for each node (OTU). All metrics indicated that candidate-core OTUs were significantly more important than non-core OTUs (Table 4 and Additional file 1: Table S10; Fig. 4 and Additional file 1: Fig. S8).

**Table 4:** Wilcoxon rank-sum test results highlighting differences in SPIEC-EASI network metrics for candidate-core and non-core taxa in field grown *Musa* spp

Metric	Candidate-core (median, 1st quartile, 3rd quartile)	Non-core (median, 1st quartile, 3rd quartile)	*W*	*P*
Betweenness centrality 411 (116, 1572)	49 (0, 298)	13,085	< 0.001	
Weighted degree	0.57 (0.3, 1.3)	0.29 (0.1, 0.6)	12,620	< 0.001
PageRank	2.95*103 (1.8*103, 5.2*103)	2.71*10–3 (8.2*10–4, 2.7*10–3)	12,717	< 0.001
Closeness centrality	4.29*105 (4.3*105, 4.3*105)	4.27*10–5 (4.2*10–5, 4.3*10–5)	12,608	< 0.001
Degree	8 (5, 17)	5 (2, 9)	11,920	< 0.001
Markov centrality	4.93*104 (4.5*104, 5.7*104)	4.37*10–4 (3.0*10–4, 5.1*10–4)	11,800	< 0.001

*P* < 0.05*, *P* < 0.01**, *P* < 0.001***

**Fig. 5:**
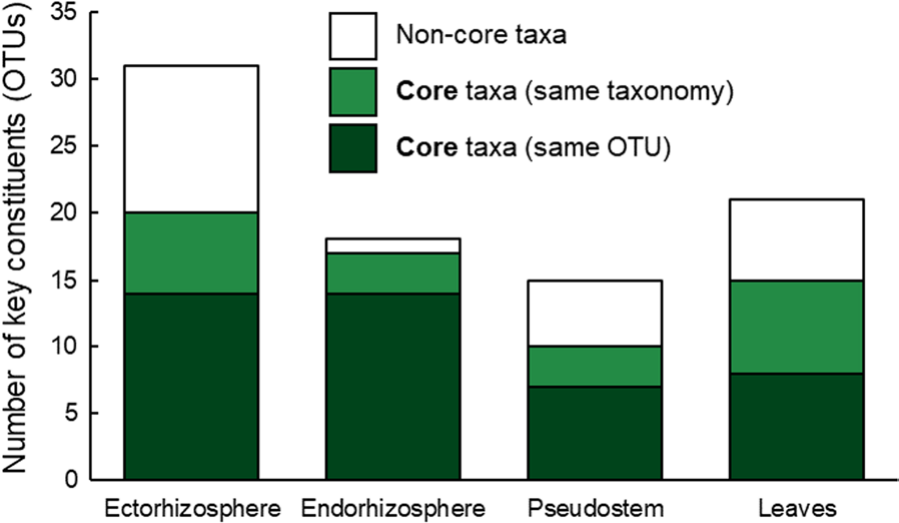
The total numbers of ‘key constituent’ OTUs in field-grown *Musa* spp. (i.e. OTUs in ≥ 50% of field-grown plants at ≥ 0.5% relative abundance) and the numbers that match core taxa identified in this study

**Fig. 6:**
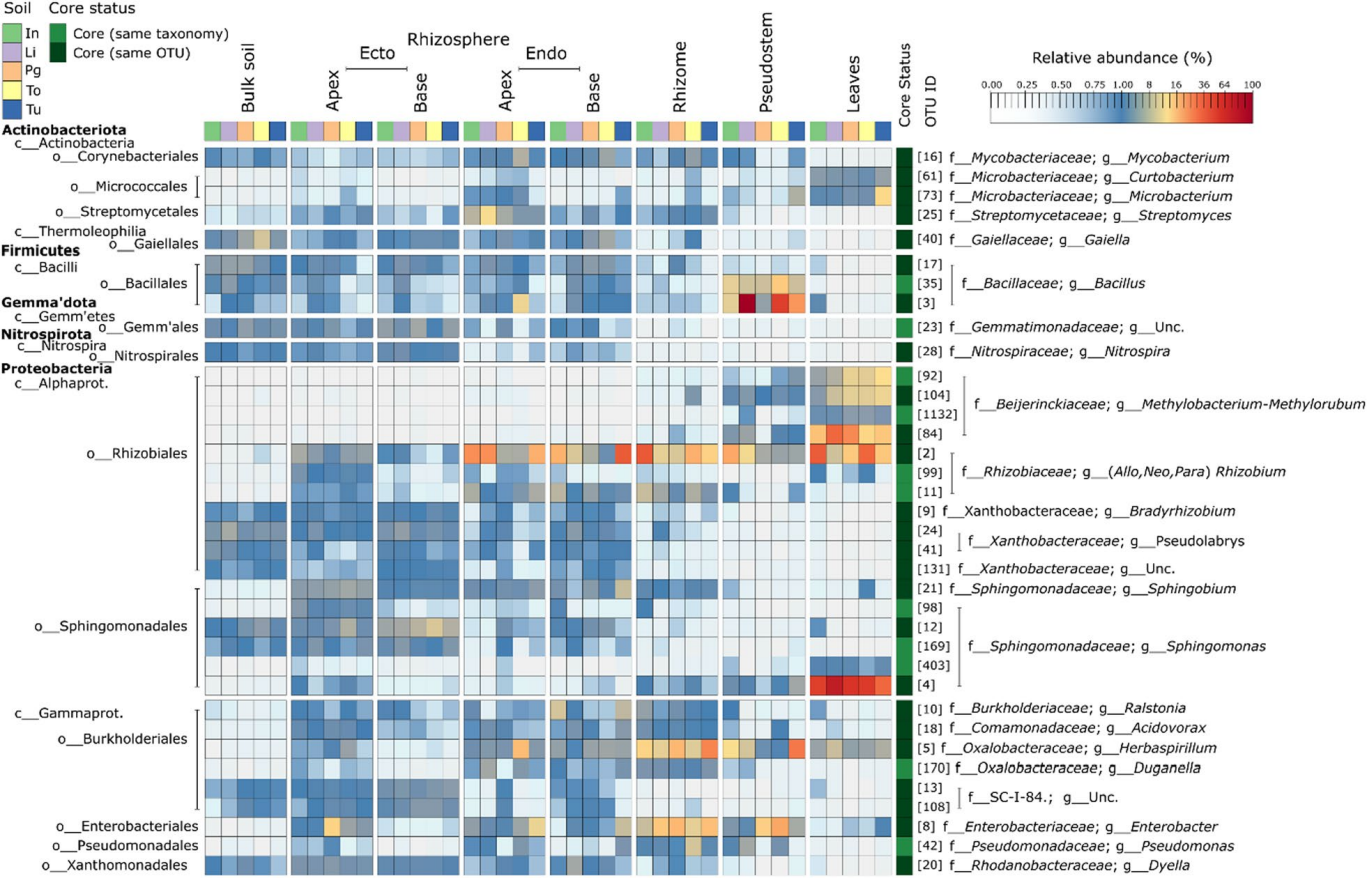
The core bacterial microbiome of *Musa* spp. The heatmap highlights the relative abundances of core OTUs in each compartment and soil for the *Musa* (AAA Group, Cavendish Subgroup) ‘Williams’ plants within the pot experiment. Each cell represents the mean of the replicates for that treatment

*Representation of candidate-core OTUs in field microbiomes* In total, 90 OTUs were classified as ‘key constituents’ of field-grown *Musa* spp. microbiomes (i.e. OTUs present in at least one plant compartment, in ≥ 50% of plants, at ≥ 0.5% relative abundance), and while representing only 1.3% of total OTUs (6775), they accounted for 42% of total sequences. Among the key constituents, 77% (36/47) of the ‘candidate-core’ taxa were present as the same OTU, or less frequently, as a close relative (i.e. an OTU with identical taxonomy; Fig. 5). These candidates were elevated to full ‘core’ status (Fig. 6 and Additional file 1: Fig. S9), while those that were not found were dropped.

### The core Musa spp. bacterial microbiome

The 36 core taxa are associated with 17 distinct genera. They include members of the Actinobacteriota, Firmicutes, Gemmatimonadota, Nitrospirota, and Proteobacteria, with notably diverse representation among the proteobacterial orders: Rhizobiales, Sphingomonadales and Burkholderiales (Fig. 6 and Additional file 1: Fig. S9; Table S11). Of a total of 6775 OTUs in field-grown plants, 62.4% were singletons or doubletons and represented only 3.2% of total sequences. The 36 core OTUs, on the other hand, represented < 0.5% of total OTUs in field-grown plants but 24.5% of total sequences, with close relatives (OTUs with the same taxonomy) representing an additional 4.0% of total OTUs and 7.4% of total sequences (Additional file 1: Fig. S10).

Within compartments, core taxa represented a larger proportion of the key constituents of field microbiomes than non-core taxa (viz. ectorhizosphere = 65%, endorhizosphere = 95%, pseudostem = 67% and leaves = 71%; Fig. 5), as well as being some of the most abundant key constituents (Additional file 1: Figs. S11–S14). Furthermore, except for OTU 61, which was absent in the genotype, Sugar (AAB), all core taxa were present in all 52 genotypes, either as the same OTU or a close relative (i.e. an OTU with identical taxonomy; Additional file 1: Figs. S10–S13). In other words, 100% of core taxa were present in 98% of genotypes, while Sugar harboured 97% of the core taxa. The representative sequences for the core and candidate-core OTUs are provided in Additional file 1: Table S11.

### Have our core OTUs been detected in other banana-related studies?

**Fig. 7:**
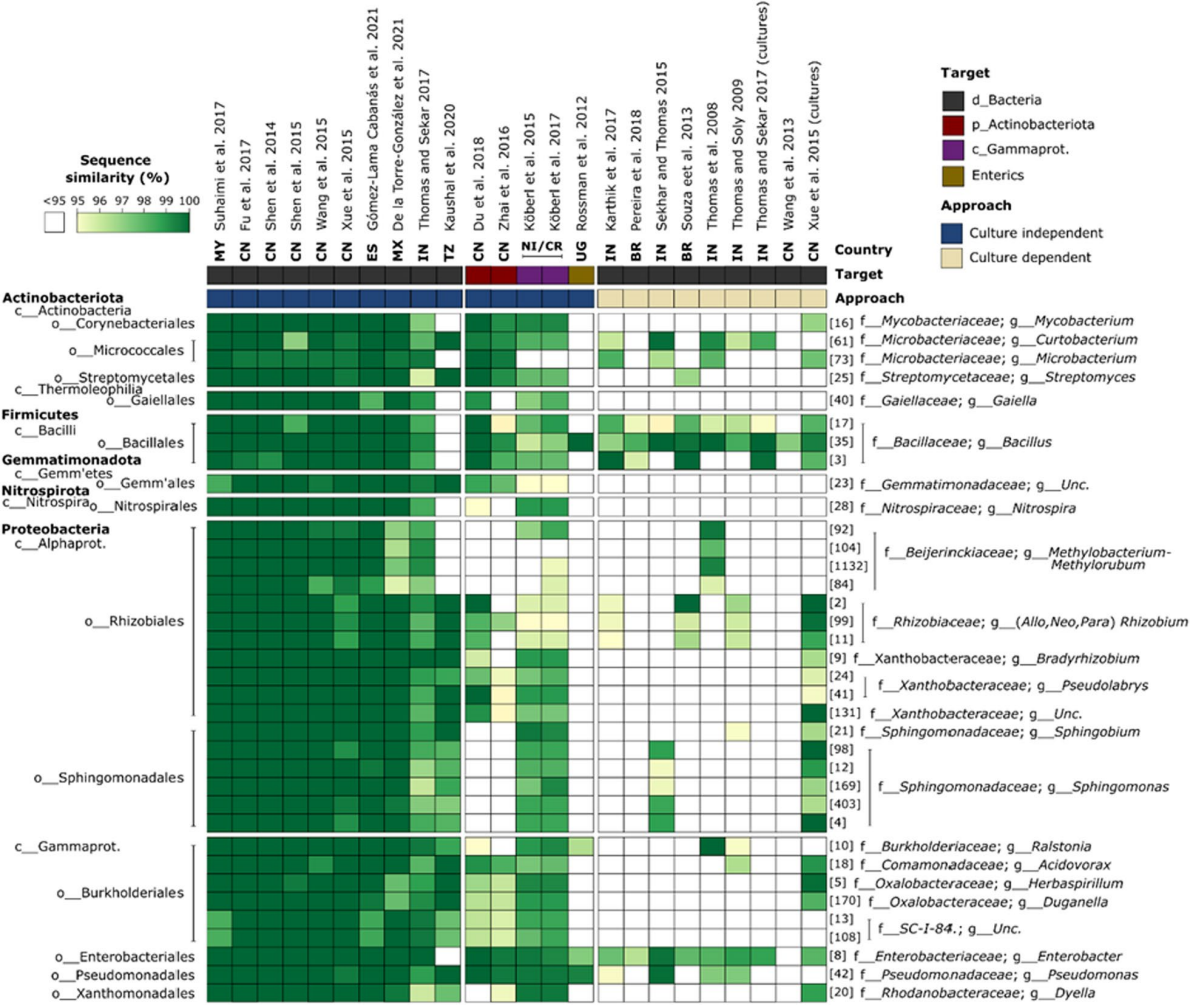
Similarity of bacterial sequences detected in 22 previous studies of banana-associated bacteria to those of the common core OTUs presented in the present study. Countries of origin are shown as two letter codes as described in the ISO 3166 international standard. Note that for two studies [50, 51], data were generated and are shown for both culture independent and dependent methods

To infer the potential geographical range of our core OTUs, we compared their sequences with those reported in 22 previous studies of banana-associated bacterial communities (Fig. 7; Additional file 1: Table S3). For the 10 culture-independent studies that characterised the whole domain, highly similar matches were observed for all populations (Fig. 7). On average, for example, 100%, ≥ 99% and ≥ 97% matches were observed 72.5%, 82.5%, and 95% of these 10 studies, respectively (Fig. 7). In contrast, fewer close matches were detected in the nine culture-dependent studies, with 13 and 15 of our core OTUs having a ≥ 97% match in zero or just one study, respectively (Fig. 7).

## Discussion

Common core bacterial taxa represent the bacterial component of a microbiome that are relatively frequent across all, or most, individuals of a host species [52]. Hence, in the context of learning how to manage crop microbiomes, they represent logical candidates for further study because they are the most likely organisms to be present. While we acknowledge that rare microbes also contribute to host function [53], we posit that it is important to first identify the most common taxa and then develop an understanding of their ecological preferences and functional traits. Here we focussed on identifying the common core bacterial taxa associated with *Musa* spp. To this end we considered it necessary to: (1) characterise bacterial communities using methods that encompass all, or most, lineages within the domain, (2) account for variation throughout the plant, and (3) consider variation associated with factors that are likely to differ between farms (e.g. edaphic conditions and cultivar selection). Hence, we sequenced ‘universal’ bacterial 16S rRNA genes amplified from the microbiomes of multiple plant compartments, soils and genotypes, in pot and field experiments.

### Musa spp. are associated with diverse bacterial communities

Our results, encompassing variation throughout the plant, highlight that banana microbiomes comprise diverse bacterial lineages, with most frequent representation within the Actinobacteriota, Firmicutes, and Proteobacteria (Fig. 3). This is in broad agreement with other studies that have identified *Musa* spp.-associated bacteria using methods that target the ‘whole’ domain, albeit these tend to have either focussed exclusively on bulk soil [54–57], or characterised single plant compartments, such as the pseudostem [58], endorhizosphere [59, 60], shoot tips [50], or ectorhizosphere [61]. In contrast, comparisons of bacterial diversity between multiple banana plant compartments tend to have focussed on specific lineages, such as the Gammaproteobacteria [24, 62, 63], shown here to represent 6–43% mean relative abundance depending on plant compartment. In the sole exception to this, where bacterial communities were characterised using ‘universal’ methods in multiple banana plant compartments (rhizosphere, roots, and corm), statistical comparisons of diversity were not possible due to a lack of replication [60]. Hence, our results greatly expand existing knowledge of the spatial distribution and structure of bacterial communities throughout banana plants.

### Bacterial diversity differs between banana plant compartments

We observed large differences in the alpha diversity (Fig. 1 and Additional file 1: Fig. S5) and composition (Fig. 2 and Additional file 1: Fig. S6) of bacterial communities between plant compartments. These differences were larger than those attributable to genotype and edaphic conditions and were remarkably similar in the pot and field experiments, which focussed on three-month old and mature plants of varying ages, respectively. Hence, *Musa* spp. appear to establish compartment-specific microbiomes from an early age that persist through to maturity. This information is useful from a management perspective and is not the case in all plants [64].

Relative to bulk soil, the compositional similarity of bacterial communities in both experiments followed the order: ectorhizosphere > endorhizosphere > pseudostem > leaves (Fig. 2 and Additional file 1: Fig. S6), but the magnitude of these changes was not even. For example, there were larger shifts in membership between ecto- and endo-rhizosphere communities, than between the ectorhizosphere and surrounding bulk soil (Additional file 1: Fig. S6). This transition, from outside to inside the roots, was also associated with the largest reductions in alpha diversity (Fig. 1 and Additional file 1: Fig. S5). These observations are consistent with studies of other plants [65–68] and likely reflect the need for bacteria to have a range of traits that enable them to enter roots and persist [69, 70]. A meta-analysis of 25 previous studies found that the endophytic bacterial communities of various plant species were dominated by Proteobacteria, Actinobacteriota, Firmicutes and Bacteroidota, while members of the Acidobacteriota and Gemmatimonadota were rare despite being common in bulk and ectorhizosphere soil [67]. These observations are commersurate with our findings for *Musa* spp. endophytes (Fig. 3 and Additional file 1: Fig. S4). Other relatively large shifts in community composition included those between the endorhizosphere, rhizome, pseudostem and leaves (Fig. 2 and Additional file 1: Fig. S6), perhaps reflecting substantial differences in environmental conditions between these habitats.

In terms of dispersal, roots are typically considered to be colonised by microorganisms from soil, while leaf communities are thought to comprise a larger proportion of taxa from aerial sources [16, 71, 72]. Our SourceTracker analyses indicated that while 81–90% of the ectorhizosphere community may be sourced from bulk soil, only 11–21% was potentially sourced from leaves (Additional file 1: Table S6). In the endorhizosphere, however, the likely contributions from leaves (37–40%) were almost as large as those from bulk soil (48–54%), suggesting that within the plant, microorganisms are relatively easily dispersed between above and belowground compartments (Additional file 1: Table S6). That said, the estimated contributions to the rhizome and pseudostem from leaves (63%) were larger than those from bulk soil (36–55%; Additional file 1: Table S6), suggesting that the rhizome, particularly towards the roots, limits microbial dispersal. Knowledge of the transmission of endophytes within banana would be useful to support the development of microbiome management approaches. For example, if core taxa could be isolated it may be possible to increase their abundances inside roots, or other compartments, via pseudostem injection – a common method for herbicide treatment on commercial banana farms.

### Musa spp. microbiomes differ between soils, immature pot plants and field-grown plants, but not genotypes

Many previous studies have demonstrated that, at least belowground, soil is the main allogenic factor influencing plant microbiomes [73–75]. Hence, it was important to determine whether differences in edaphic properties influenced *Musa* spp. microbiomes, and if so, to consider only taxa present in all soils tested for designation as members of the common core. As such, we grew bananas in five distinct soils from Australia’s main production area and profiled their microbiomes. We found that, with the exception of leaves, bacterial communities in all plant compartments differed significantly between soils. This highlights that the dominant bacteria associated with *Musa* spp. in one area may not be present in another due to differences in edaphic conditions. For this reason, we only considered bacteria that were strongly represented in all soils tested when selecting candidate core taxa.

Given that a wide variety of bananas are grown around the world [10], it was also important to consider whether differences in genotype influence *Musa* spp. microbiomes. Hence, we compared the microbiomes of *Musa* (AAA Group, Cavendish Subgroup) ‘Williams’, *Musa* (AAB Group, Pome Subgroup) ‘Lady Finger’, and *Musa* (AAAB Group, Prata Anã x SH-3142) 'Goldfinger'. Despite, differences in ploidy and genome composition between these cultivars [76], the diversity of their bacterial communities did not differ significantly irrespective of plant compartment. In some plant species, different genotypes have been shown to harbour distinct bacterial communities [77–79]. However, this is not always the case [80], and as a rule of thumb, host phylogenetic distance tends to be negatively associated with the similarity of root microbiomes [6]. Hence, our observation that bacterial diversity did not differ significantly between *Musa* spp. genotypes, may reflect the fact that commercial *Musa* spp. are clonally propagated and closely related to their wild relatives [76, 81].

As the microbiomes of pot- and field-grown can vary [64, 77, 82, 83], we assessed whether the candidate-core taxa identified in our pot experiment were relevant in field-grown plants. We found that eleven candidate-core taxa were not represented in field-grown plants, which could result from a range of factors. For example, glasshouses may physically limit the dispersal of environmental microbes [84], particularly from aerial sources, which may explain why the alpha diversity of above-ground compartments in field-grown plants was larger than in our potted plants (Additional file 1: Fig. S6). Furthermore, our field grown plants were typically mature and in flower, whereas those in our pot experiments were immature. Plant developmental stage has been shown to strongly influence microbiomes due associated changes in rhizodeposition, host metabolism and immunity [64]. In addition, community succession can influence plant microbiomes through developmental stages, as pioneer species are overtaken by later successional species [85, 86]. Despite these differences, 36 of the candidate-core taxa identified in the pot study persistently associated with *Musa* spp. in both pot and field conditions.

### Defining and refining the core bacterial microbiome of Musa spp.

Based on the findings of our pot experiments, we identified 47 OTUs that were relatively frequent in at least one compartment across most of the plants grown in each soil. As microbiomes can differ between pot and field-grown plants [82, 83], we considered these OTUs as ‘candidates’ rather than immediately designating them as common core taxa. Full core status was assigned only after confirming that they were relatively frequent in at least one compartment across most of the field-grown plants characterised in our survey of the Australian Banana Germplasm Collection. Of the 47 candidates, 36 OTUs were confirmed to be frequent in most plants and were elevated to full core status (Additional file 1: Figs. S10–S13). In the endorhizosphere, for example, 95% of dominant OTUs were listed as members of the common core (Fig. 5), and of the 52 *Musa* spp. genotypes surveyed, 51 contained all core OTUs, with only one missing in the other genotype.

### Core OTUs have close banana-associated relatives around the world

By comparing the sequences of our core OTUs with those detected in 22 previous studies of banana-associated bacteria we found that they have close relatives in Brazil, China, Costa Rica, the Canary Islands, India, Malaysia, Mexico, Nicaragua, Tanzania, and Uganda. Hence, these findings indicate that our core taxa are pervasively associated with *Musa* spp. across steep environmental gradients spanning different time points, continents, genotypes, plant ages, climates, and edaphic factors. This raises the possibility that they play important roles in the regulation of host function, as has been suggested in other plant microbiome studies [7–9]. To test this hypothesis empirically, the functional responses of *Musa* spp. to changes in the frequencies and/or activities of core taxa need to be measured. This is beyond the scope of our current study; however, in the following discussion we present evidence from network analyses and inferences from the literature that support the notion that our core taxa influence host fitness.

### Evidence that core OTUs are important ‘hub species’

Network analyses indicated that common core OTUs were significantly more interconnected than non-core OTUs and could be considered as ‘microbial hubs’ [87] (Table 4). While these apparent interactions may be indirect, current evidence indicates that ‘hub species’ are important for host fitness and can mediate interactions between the plant and the microbiome [87]. Hence, their loss may render the host more susceptible to disease and compromise resource acquisition [88]. However, there are examples where hub species negatively impact their host. For example, while enhancing plant nutrient uptake, mycorrhizal fungi can inadvertently promote plant-parasitic nematodes by disarming plant defences [89]. Similarly, while facilitating efficient energy harvest, certain gut microbes can promote obesity in humans [90].

### Associations of core taxa with host fitness

As all plants in our study lacked symptoms of disease or signs of abnormal pest pressure, the common core OTUs may be considered representative of ‘healthy’ banana plants. Interestingly, circumstantial evidence from the literature supports that changes in the relative abundances of populations closely related to our common core influence plant health (Additional file 1: Table S12). For example, in a comparison of the pseudostems of healthy and bacterial wilt affected bananas, all of our core pseudostem taxa were found in healthy plants, but only three were detected in sick plants [58]. Furthermore, rotating banana production with pineapple [55], or chilli [91] has been found to reduce the severity of Fusarium Wilt of Banana (FWB) while increasing the relative abundances of genera that contain close relatives of our common core: i.e. *Bradyrhizobium,* or *Gemmatimonas*, *Pseudomonas*, *Sphingobium,* and *Sphingomonas*, respectively. Direct addition of isolates that are closely related to the core has also been observed to protect bananas against diseases. For example, *Pseudomonas* spp. have been shown to lessen the severity of FWB [59, 92–94] and Banana bunchy top disease [95]. Similarly, the addition of *Bacillus* spp. to soil has been shown to reduce the severity of FWB [57, 96–99], with concomitant increases in the relative abundances of *Gemmatimonas*, *Sphingomonas*, *Rhizobium* and/or *Pseudolabrys* populations [100]. Lastly, many studies have demonstrated suppression of *Foc* in culture by close relatives of the common core isolated from banana [101], including members of the *Bacillus* [102, 103], *Pseudomonas* [59, 104, 105] *Rhizobium* [104], and *Streptomycetes* [106].

Despite being representative of ‘healthy’ banana plants, some members of the common core are closely related to lineages that harbour devastating *Musa* spp. pathogens. For example, OTU10 is a member of the genus *Ralstonia*, which includes the causal agents of Moko/Bugtok disease (*Ralstonia solanacearum*) and banana blood disease (*Ralstonia syzygii* ssp. *celebensis*). In addition, OTU8 and OTU20 are members of the *Enterobacteriaceae* and *Xanthomonadales*, which include the causal agents of *Erwinia*-associated diseases (i.e. *Erwinia carotovora* ssp. *carotovora*; *E. chrysanthemi*, and *Dickeya paradisiaca*) and Xanthomonas wilt (*Xanthomonas campestris* pv. *musacearum*) [107]. While these populations may be latent pathogens [108], bacterial genera frequently contain species with non-pathogenic and pathogenic strains [109–111] – differentiated by only a small number of virulence genes [108]. Hence, given the persistently strong associations between common core taxa and their host, it is logical that cooperative symbioses will be undermined, from time-to-time, by ‘cheaters’, such as parasites and pathogens [112].

Evidence suggests that common core taxa may also influence other aspects of banana plant fitness. Plant growth promotion, for example, has frequently been observed in bananas inoculated with close relatives of the common core originally isolated from *Musa* spp., such as *Bacillus* spp. [51, 113, 114], *Enterobacter* spp. [115], *Pseudomonas* spp. [116] and *Rhizobium* spp. [117, 118]. Furthermore, genome sequences and in vitro lab assays have revealed that these, and other close relatives of the common core, harbour genes and exhibit phenotypes that are associated with multiple approaches to promote plant growth, enhance plant stress tolerance, and interact with other organisms [59, 70, 105, 118–121]. *Rhizobium* spp. isolated from banana, for example, have been reported to produce the auxin phytohormone indole-3-acetic acid, fix nitrogen, and solubilise phosphate [118].

## Conclusion

Our findings have meaningful implications for the development of strategies to manage *Musa* spp. microbiomes. We have demonstrated that while the bacterial communities associated with banana are highly similar across genotypes, they can vary greatly between locations due to differences in edaphic conditions. Hence, our findings support the notion that research efforts to maximise plant productivity by controlling the abundances and activities of their symbionts, are best focussed on core microbes, as they are most likely to be present. Critically, we have identified 36 common core OTUs that share 100% sequence similarity with bacteria detected in bananas grown in other parts of the world. These core bacteria were found in pot and field grown banana plants, irrespective of soil and genotype, and were identified as highly interconnected ‘hubs’ within networks – a characteristic shown to be indicative of microbes that influence host fitness in studies of other plants. Circumstantial evidence from the literature demonstrates that close relatives of the common core help to reduce banana diseases and promote growth. However, to justify a focus of research efforts on this small but frequently encountered subset of taxa, it is critical to experimentally verify these associations. To do this, it is necessary to measure the functional responses of *Musa* spp. to changes in the frequencies of core taxa. Such changes may be achieved by manipulating soil properties (e.g. pH [122]), or on-farm practices (e.g. rotation [91]). However, while these approaches may represent useful levers for future microbiome managers, current knowledge of their effects on the abundances of core taxa is sparse, and their lack of specificity represents a major obstacle to establishing the causality of plant–microbe interactions. Another approach may be to manipulate the frequencies of core taxa through their cultivation and subsequent addition to *Musa* spp. in gnotobiotic experiments, either alone, or as synthetic communities (SynComs [123, 124]). The controlled nature of these studies would help to establish causality and to identify the mechanisms of interaction between banana and their core bacteria. In this regard, we would like to point out that highly similar matches to some of our core OTUs have already been isolated in previous studies and may be suitable for experimentation (Fig. 7). Having identified a robust list of banana-associated core bacteria, we hope that this contribution will accelerate the acquisition of knowledge that is most relevant to the development of effective *Musa* spp. microbiome management practices.

## Supplementary Information

**Additional file 1**: **Table S1**. The locations and basic properties of the five soils used to grow Musa spp. in the pot experiment of this study. **Table S2**. Musa spp. genotypes included in our survey of field-grown plants in the Australian Banana Germplasm Collection. **Table S3**. Studies included in a meta-analysis of the prevalence of core microbes identified in this study in other studies of bacteria associated with Musa spp. **Fig. S1**. A map showing, in black, countries included in the meta-analysis of the bacterial microbiome of *Musa* spp. Countries in grey are major producers (FAOSTAT, 2022) for which data were not available and were not included in meta-analysis. **Table S4**. The impact of soil, genotype and plant compartment on alpha diversity metrics assessed by ANOVA from the bacterial microbiome of *Musa *spp. These results derive from our pot experiment which included five distinct soils, three Musa spp. genotypes, and eight compartments, each with 10 replicates. **Table S5**. The impact of soil, genotype and plant compartment on bacterial community composition as represented by Weighted UniFrac distances using PERMANOVA. These results derive from our pot experiment which included five distinct soils, three *Musa* spp. genotypes, and eight compartments, each with 10 replicates. **Table S6**. Average percentage similarity plus/minus the standard deviation of various *Musa* spp. plant compartments. Percentages were produced using a Bayesian approach implemented through SourceTracker. **Table S7**. The influence of soil on the alpha diversity of bacterial communities within each compartment, as assessed by ANOVA. The results are for the Musa (AAA Group, Cavendish Subgroup) ‘Williams’ plants grown in five distinct soils in our pot experiment. **Table S8**. The influence of soil on the composition of bacterial communities, as represented by Weighted UniFrac distances, within compartments as assessed using PERMANOVA. The results are for the Musa (AAA Group, Cavendish Subgroup) ‘Williams’ plants grown in five distinct soils in our pot experiment. **Fig. S2**. Venn diagrams to show the numbers of shared candidate-core OTUs between Musa (AAA Group, Cavendish Subgroup) ‘Williams’ grown in five distinct soils (Pg, Tu, In, Li, and To) within compartments. Candidate-core OTUs are those that were present in ≥ 50% of the ten replicates within each treatment combination at a mean relative abundance of ≥ 0.5%. Core OTUs are those that were shared between all soils within each compartment. These results are from our pot experiment. **Fig. S3**. Heatmaps showing the number of soils in which each OTUs was considered a ‘candidate-core’ population on a per compartment basis. The results are for the Musa (AAA Group, Cavendish Subgroup) ‘Williams’ plants grown in five distinct soils in our pot experiment. **Fig. S4**. The ‘candidate’ core bacterial microbiome of *Musa* spp. The heatmap highlights the relative abundances of core OTUs in each compartment and soil for the Musa (AAA Group, Cavendish Subgroup) ‘Williams’ plants within the pot experiment. Each cell represents the mean of the replicates for that treatment. OTUs in grey indicate ‘candidates’ that were not found in field-grown plants and were subsequently dropped. Those with black text were found in field plants and were elevated to full core status. **Table S9**. OTUs found as candidate-core associated with Musa (AAA Group, Cavendish Subgroup) ‘Williams’ in plants grown in one to five soils or as key constituents in the field- grown *Musa* spp. **Fig. S5**. The mean relative frequencies of bacterial classes associated with each compartment in field-grown *Musa *spp. Within each phylum, classes represented at < 1% mean relative abundance are grouped as other. **Fig. S6**. Numbers of observed bacterial OTUs associated with different *Musa* spp. compartments and settings (pot vs. field). Error bars represent standard errors of the means. The letters indicate treatments that differ across soils according to estimated marginal means post hoc tests with Bejamini-Hochberg corrections. Members of the same groupings share the same letter. **Fig. S7**. A Principal Coordinate Analysis (PCA) ordination highlighting differences in the composition of bacterial communities (Hellinger transformed OTUs) associated with pot and field-grown Musa spp. in different plant compartments. Points representing field samples are larger than those representing pot samples. The ellipses represent standard deviations of the group centroids. **Table S10**. SPIEC-EASI network centrality metrics for core OTUs in field-grown Musa spp. **Fig. S8**. SPIEC-EASI network graphs showing the co-occurrences of core and non-core bacteria in field-grown *Musa* spp. Nodes are coloured by core status and size is positively associated with a range of centrality metrics. Edge colours represent positive (blue) and negative (red) associations between taxa. Edge width is positively associated with the coefficient for the co-occurrence between the taxa. The node layout in each graph is the same as in Figure 4, which shows the OTU IDs. **Fig. S9**. The common core bacterial microbiome of Musa spp. Blue tiles highlight which OTUs are core within each plant compartment. **Fig. S10**. Percentage of total bacterial sequences attributable to core OTUs and non-core OTUs within Musa spp. grown in the field or in pots containing different soils (In, Li, Pg, To, Tu). **Fig. S11**. A heatmap representing the mean relative abundances of key constituent OTUs in the ectorhizosphere of field-grown *Musa* spp. (i.e. present at ≥0.5% relative abundance in ≥50% of samples). The green squares indicate taxa that are members of the core microbiome. **Fig. S12**. A heatmap representing the mean relative abundances of key constituent OTUs in the endorhizosphere of field-grown *Musa* spp. (i.e. present at ≥ 0.5% relative abundance in ≥ 50% of samples). The green squares indicate taxa that are members of the core microbiome. **Fig. S13**. A heatmap representing the mean relative abundances of key constituent OTUs in the pseudostem of field-grown *Musa* spp. (i.e. present at ≥ 0.5% relative abundance in ≥ 50% of samples). The green squares indicate taxa that are members of the core microbiome. **Table S11**. Representative sequences the 47 candidate-core OTUs identified in the Musa (AAA Group, Cavendish Subgroup) ‘Williams’ plants grown in the five distinct soils in our pot experiment. OTUs that were elevated to full core-status are marked as core in bold text. **Fig. S14**. A heatmap representing the mean relative abundances of key constituent OTUs in the leaves of field-grown *Musa* spp. (i.e. present at ≥ 0.5% relative abundance in ≥ 50% of samples). The green squares indicate taxa that are members of the core microbiome. **Table S12**. Examples from the literature where core and candidate-core taxa identified in this study have been reported to be important to Musa spp. plant fitness or associated with the plant.

## Data Availability

The 16S rRNA gene amplicon sequences associated with this study have been deposited in the NCBI SRA under accession: PRJNA729168.
