# Supplementary material for: The core bacterial microbiome of banana (Musa spp.)
Source: Environ Microbiome. 2022 Sep 8;17:46. doi: 10.1186/s40793-022-00442-0 (PMC9461194; doi:10.1186/s40793-022-00442-0)
Supplement: Supplementary file 1 — Additional file 1: Table S1. The locations and basic properties of the five soils used to grow Musa spp. in the pot experiment of this study. Table S2. Musa spp. genotypes included in our survey of field-grown plants in the Australian Banana Germplasm Collection. Table S3. Studies included in a meta-analysis of the prevalence of core microbes identified in this study in other studies of bacteria associated with Musa spp. Fig. S1. A map showing, in black, countries included in the meta-analysis of the bacterial microbiome of Musa spp. Countries in grey are major producers (FAOSTAT, 2022) for which data were not available and were not included in meta-analysis. Table S4. The impact of soil, genotype and plant compartment on alpha diversity metrics assessed by ANOVA from the bacterial microbiome of Musa spp. These results derive from our pot experiment which included five distinct soils, three Musa spp. genotypes, and eight compartments, each with 10 replicates. Table S5. The impact of soil, genotype and plant compartment on bacterial community composition as represented by Weighted UniFrac distances using PERMANOVA. These results derive from our pot experiment which included five distinct soils, three Musa spp. genotypes, and eight compartments, each with 10 replicates. Table S6. Average percentage similarity plus/minus the standard deviation of various Musa spp. plant compartments. Percentages were produced using a Bayesian approach implemented through SourceTracker. Table S7. The influence of soil on the alpha diversity of bacterial communities within each compartment, as assessed by ANOVA. The results are for the Musa (AAA Group, Cavendish Subgroup) ‘Williams’ plants grown in five distinct soils in our pot experiment. Table S8. The influence of soil on the composition of bacterial communities, as represented by Weighted UniFrac distances, within compartments as assessed using PERMANOVA. The results are for the Musa (AAA Group, Cavendish Subgroup) ‘Williams’ plants [file 40793_2022_442_MOESM1_ESM.pdf]

Supplementary information:

The Core Bacterial Microbiome of Banana (*Musa* spp.)

Henry W. G. Birt[1], Anthony B. Pattison[1,2], Adam Skarshewski[1], Jeff Daniells[2], Anil Raghavendra[1], Paul G. Dennis[1*]

[1]*School of Earth and Environmental Sciences, The University of Queensland, Brisbane, QLD 4072, Australia;* [2]*Department of Agriculture and Fisheries, Centre for Wet Tropics Agriculture, 24 Experimental Station Road, South Johnstone, QLD 4859, Australia;*

**Correspondence*: p.dennis@uq.edu.au

Contents:

Table S1 Locations and properties of pot experiment soils……………………………………….3

Table S2 *Musa* spp. genotypes included in our field survey……………………………………...4

Table S3 Studies included in meta-analysis of *Musa* spp. bacteria………………………...5-10

Fig. S1 Map of countries included in meta-analysis of *Musa* spp. bacteria…………………...11

Table S4 Impact of soil, genotype and plant compartment on alpha diversity………………..12

Table S5 Impact of soil, genotype and plant compartment on Weighted UniFrac……………13

Table S6 Similarity of *Musa* spp. plant compartments……………………………………..…14

Table S7 Influence of soil on alpha diversity within compartment…………………………...15

Table S8 Influence of soil on Weighted UniFrac within compartment……………………….16

Fig. S2 Numbers of shared candidate-core OTUs between soils……………………………...17

Fig. S3 Number of soils in which OTUs were considered ‘candidate-core’…………………..18

Fig. S4 The ‘candidate’ core bacterial microbiome of *Musa* spp……………………………..19

Table S9 Candidate core OTUs listed by prevalence…………………………………………20

Fig. S5 Stacked bar chart of bacterial classes in field-grown *Musa* spp………………………21

Fig. S6 Bacterial richness in pot vs. field plants……………………………………………22

Fig. S7 PCA of bacterial community composition in pot vs. field plants……………………..23

Table S10 Network metrics for core OTUs in field-grown *Musa* spp………………………...24

Fig. S8 Network graphs of core and non-core bacteria in field-grown *Musa* spp……………..25

Fig. S9 Common core OTUs in each plant compartment……………………………………..26

Fig. S10 Proportion of sequences represented by core in pot and field grown plants…………27

Fig. S11 Key constituent OTUs in the ectorhizosphere of field-grown *Musa* spp…………….28

Fig. S12 Key constituent OTUs in the endorhizosphere of field-grown *Musa* spp……………29

Fig. S13 Key constituent OTUs in the pseudostem of field-grown *Musa* spp………………...30

Fig. S14 Key constituent OTUs in the leaves of field-grown *Musa* spp………………………31

Table S11 Sequences core and candidate-core OTUs…………………………………….32-37

Table S12 Studies linking *Musa* spp. and host fitness to core taxa……………………….38-46

References……………………………………………………………………………..47-51

Table S1 The locations and basic properties of the five soils used to grow *Musa* spp. in the pot experiment of this study.

**Parameter**	**Innisfail**	**Liverpool**	**Pin Gin**	**Tolga**	**Tully**
Latitude (°S)	17.485122	17.454604	17.592257	17.010557	17.481890
Longitude (°E)	145.859047	145.864289	145.833088	145.527065	145.858678
Texture	Clay Loam	Clay Loam	Clay	Clay	Clay loam
Clasification	Brown Dermosol	Orthic Tenosol	Red Ferrosol	Red Ferrosol	Brown Dermosol
Clay (%)	27	26	39	56	28
pH	6.7	7.1	7.1	5.6	6.6
Total C (%)	2.067	1.558	2.417	2.843	2.837
Total N (%)	0.180	0.143	0.212	0.190	0.210
C:N	11.2	10.8	11.5	14.9	13.5

Table S2 *Musa* spp. genotypes included in our survey of field-grown plants in the Australian Banana Germplasm Collection.

Cultivar	Subgroup/Species & Subspecies	Genome
845	*Musa acuminata* ssp. *malaccensis*	AA
846	*Musa acuminata* ssp. *malaccensis*	AA
848	*Musa acuminata* ssp. *malaccensis*	AA
850	*Musa acuminata* ssp. *malaccensis*	AA
851	*Musa acuminata* ssp. *malaccensis*	AA
852	*Musa acuminata* ssp. *malaccensis*	AA
Agutay	*Musa acuminata* ssp. *errans*	AA
Musa balbisiana	*Musa balbisiana*	BB
Blue Java	Ney Mannan	ABB
Bluggoe	Bluggoe	ABB
Borneo	*Musa acuminata* ssp. *microcarpa*	AA
Calcutta	*Musa acuminata* ssp. *burmannicoides*	AA
Cam 020	Not available	--
Williams	Cavendish	AAA
Ducasse	Pisang Awak	ABB
Dwarf French Plantain	Plantain	AAB
Dwarf Nathan	Cavendish	AAA
FHIA-02	Cavendish hybrid/Pome hybrid	AAAB
FHIA-03	Not available	AABB
FHIA-17	Highgate hybrid	AAAA
FHIA-18	Pome hybrid	AAAB
FHIA-23	Highgate hybrid	AAAA
FHIA-25	Not available	AAB
GCTCV 218 (Formosana)	Cavendish	AAA
GCTCV 105	Cavendish	AAA
GCTCV 119	Cavendish	AAA
GCTCV 215	Cavendish	AAA
GCTCV 217	Cavendish	AAA
Goldfinger	Pome hybrid	AAAB
Grande Naine	Cavendish	AAA
Gros Michel	Gros Michel	AAA
Highgate	Gros Michel	AAA
Igisahira Gsanzwe	Mutika/Lujugira	AAA
Lakatan	Lakatan	AAA
Lady Finger	Pome	AAB
Ney Poovan	Ney Poovan	AB
Niukin	Pisang Jari Buaya	AA
Pisang Mas	Sucrier	AA
PA 03.22	Pome hybrid	AAAB
Pa Payang	*Musa acuminata* ssp. *siamea*	AA
Pacific Plantain	Maoli/Popoulu	AAB
Pahang	*Musa acuminata* ssp. *malaccensis*	AA
Pisang Ceylan	Mysore	AAB
Pisang Gajih Merah	Saba	ABB
Pisang Raja	Pisang Raja	AAB
Red Dacca	Red	AAA
Santa Catarina Prata	Pome	AAB
SH-3142	Not available	AA
SH-3362	Not available	AA
Sugar	Silk	AAB
Yangambi Km5	Ibota	AAA
Zebrina	*Musa acuminata* ssp. *zebrina*	AA

Table S3 Studies included in a meta-analysis of the prevalence of core microbes identified in this study in other studies of bacteria associated with *Musa* spp.

Study	Method	Plant compartments	Cultivar	Taxonomic focus	Country	Study description	Title	Accession
De la Torre-González et al. (2021) [1]	Culture independent (Illumina MiSeq)	Ectorhizosphere, bulk soil	*Musa acuminata* (cultivar not given)	Bacteria	Mexico	Examine how different climate, soils, and crop management practices influence bacterial diversity in the banana plant microbiome	Response to edaphoclimatic conditions and crop management of the bacterial microbiome of *Musa acuminata* rhizosphere profiled by 16S rRNA gene amplicon sequencing	PRJNA673638
Du et al. (2018) [2]	Culture independent (Illumina MiSeq)	Pseudostem	Giant Cavendish Baxi	Actinobacteriota	China	The use of Actinobateria specific primers to examine the endophytic communities from Giant Cavendish (AAA) shoot tips.	Illumina-based sequencing analysis directed selection for actinobacterial probiotic candidates for banana plants	SRP062458
Fu et al. (2017) [3]	Culture independent (454)	Ectorhizosphere	Brazil	Bacteria	China	The effect of a biocontrol on the rhizosphere community of field grown banana plants.	Inducing the rhizosphere microbiome by biofertilizer application to suppress banana Fusarium wilt disease	SRP070868

Gómez-Lama Cabanás et al. (2021) [4]	Culture independent (Illumina MiSeq)	Endorhizosphere	Pequeña Enana (synonymous with Dwarf Cavendish)	Bacteria/fungi	Spain (Canary Islands)	Examine microbial community diversity in mother and sucker plants across various farm in the Canary Islands.	The banana root endophytome: differences between mother plants and suckers and evaluation of selected bacteria to control *Fusarium oxysporum* f. sp. *cubense*	MT445188-MT445196
Karthik et al. (2017) [5]	Culture dependent (Platform unknown)	Leaf	Rasthali, Hill banana, Co1, Nattu Poovan and Red banana	Bacteria	India	Leaf endophytes of field grown Rasthali, Hill banana, Co1, Nattu Poovan and Red banana plants.	Endophytic bacteria associated with banana cultivars and their inoculation effect on plant growth	KF036177 - KF036193
Kaushal et al. (2020) [6]	Culture independent (Illumina, specific platform unknown)	Ectorhizosphere, endorhizosphere, rhizome (corm)	Sukari Ndizi (*Musa* subgroup AAB)	Bacteria	Tanzania	Assess the diversity of bacterial microbiome of banana plants with and without Fusarium Wilt symptoms	Unlocking the microbiome communities of banana (*Musa* spp.) under disease stressed (fusarium wilt) and non-stressed conditions	PRJNA493905 and PRJNA494050
Köberl et al. (2015) [7]	Culture independent (Illumina MiSeq)	Ectorhizosphere, endorhizosphere, pseudostem, leaves	Gros Michel	Gammaproteobacteria	Costa Rica and Nicaragua	Gammaproteobacterial communities found in the roots, pseudostem and leaves of field-grown plants.	Agroforestry leads to shifts within the gammaproteobacterial microbiome of banana plants cultivated in Central America	PRJEB8107

Köberl et al. (2017) [8]	Culture independent<br>(Illumina MiSeq)	Ectorhizosphere, endorhizosphere, pseudostem, leaves	Gros Michel	Gammaproteobacteria	Costa Rica and Nicaragua	Gammaproteobacterial communities found in the roots, pseudostem and leaves of field-grown Fusarium wilt affected plants.	Members of Gammaproteobacteria as indicator species of healthy banana plants on Fusarium wilt-infested fields in Central America	PRJEB12550
Pereira et al. (2018) [9]	Culture dependent<br>(Sanger)	Endorhizosphere	Prata Anã (AAB)	Bacteria	Brazil	Root communities from field grown Prata Anã (AAB) banana.	Characterization and activity of endophytic bacteria from 'Prata Anã' banana crop (*Musa* sp., AAB)	KX189587 - KX189625
Rossman et al. (2012) [10]	Culture dependent and indepedent<br>(Culture based 16S sequences, DGGE, 454 Pyrosequencing, FISH, qPCR)	Bulk soil ectorhizosphere, pseudostem exterior	Unknown	Bacteria/Fungi<br>Enteric sequences	Uganda	Examination of soil and pseudostem of field-grown banana.	Banana-associated microbial communities in Uganda are highly diverse but dominated by *Enterobacteriaceae*	HE588041 - HE588079
Shen et al. (2014) [11]	Culture independent<br>(454)	Bulk soil	Brazil	Bacteria	China	The effect of a biocontrol on the bulk soil bacterial community of field grown banana plants.	Deep 16S rRNA pyrosequencing reveals a bacterial community associated with banana fusarium wilt disease suppression induced by bio-organic fertilizer application	DRA001282

Shen et al. (2015) [12]	Culture independent (454)	Bulk soil	Brazil	Bacteria	China	Comparing wilt suppressive/susceptible	Soils naturally suppressive to banana Fusarium wilt disease harbor unique bacterial communities	DRA002235
Souza, S. A. et al. (2013) [13]	Culture dependent (ABI-PRISM 3100 GA)	Endorhizosphere	Prata Anã (AAB)	Bacteria	Brazil	Root communities from field grown Prata Anã (AAB) banana.	Endophytic bacterial diversity in banana 'Prata Anã' (*Musa* spp.) roots	JQ979307 - JQ979408
Suhaimi et al. (2017) [14]	Culture independent (Illumina MiSeq)	Pseudostem	Nipah	Bacteria	Malaysia	Pseudostem from healthy and bacterial wilt effected field grown Nipah banana plants.	Diversity of microbiota associated with symptomatic and non-symptomatic bacterial wilt-diseased banana plants determined using 16S rRNA metagenome sequencing	SRP056352
Thomas et al. (2008) [15]	Culture dependent (Platform unknown)	Pseudostem	Grand Naine	Bacteria	India	Examination of field grown Grand Naine (AAA) shoot tips.	Identification of culturable and originally non-culturable endophytic bacteria isolated from shoot tip cultures of banana cv. Grand Naine	Various (see article)
Thomas & Soly, (2009) [16]	Culture dependent (Platform unknown)	Pseudostem	Grand Naine	Bacteria	India	Examination of field grown Grand Naine (AAA) shoot tips.	Endophytic bacteria associated with growing shoot tips of banana (*Musa* sp.) cv. Grand Naine and the affinity of endophytes to the host	EU518689-EU518713 & FJ447493-FJ447497

Thomas & Sekhar (2017) [17]	Culture independent and dependent (Illumina MiSeq)	Pseudostem	Grand Naine	Bacteria	India	Examination of field grown Grand Naine (AAA) shoot tips.	Cultivation versus molecular analysis of banana (*Musa* sp.) shoot-tip tissue reveals enormous diversity of normally uncultivable endophytic bacteria	KT944215 - KT944251, KT946872 - KT946901, SRR2664411, SRR2664412, SRR3646184, SRR3646239
Sekhar & Thomas (2015) [18]	Culture dependent (Platform unknown)	Pseudostem	Grand Naine	Bacteria	India	Examining bacteria present in banana plant shoot tips	Isolation and identification of shoot - tip associated endophytic bacteria from banana cv. Grand Naine and testing for antagonistic activity against *Fusarium oxysporum* f. sp. *cubense*	KP798811 - KP798857
Wang et al. (2013) [19]	Culture dependent (Platform unknown)	Bulk soil	Unknown	Bacteria	China	Isolation of suppressive strains from the rhizosphere of banana plants grown in a Fusarium Wilt suppressive field	Effects of novel bioorganic fertilizer produced by *Bacillus amyloliquefaciens* W19 on antagonism of Fusarium wilt of banana	JQ740889
Wang et al. (2015) [20]	Culture independent (454)	Bulk soil	Unknown	Bacteria/Fungi	China	Field soil from a field in which banana was grown after rotation with pineapple.	Pineapple-banana rotation reduced the amount of *Fusarium oxysporum* more than maize-banana rotation mainly through modulating fungal communities	DRA002471

Xue et al. (2015) [21]	Culture independent and dependent (454)	Ectorhizosphere	Brazil	Bacteria	China	Roots from field grown Brazil (AAA) banana affected by Fusarium wilt and then either treated with a biocontrol or not.	Manipulating the banana rhizosphere microbiome for biological control of Panama disease.	KJ600797 - KJ600996 & SRR1185515-SRR1185517
Zhai et al. (2016) [22]	Culture independent (Illumina MiSeq)	Pseudostem	Giant Cavendish Baxi	Actinobacteriota	China	The use of Actinobacteria specific primers to examine the root endophytes of field grown plants	A new approach to analyzing endophytic actinobacterial population in the roots of banana plants (*Musa* sp., AAA)	SRP061867

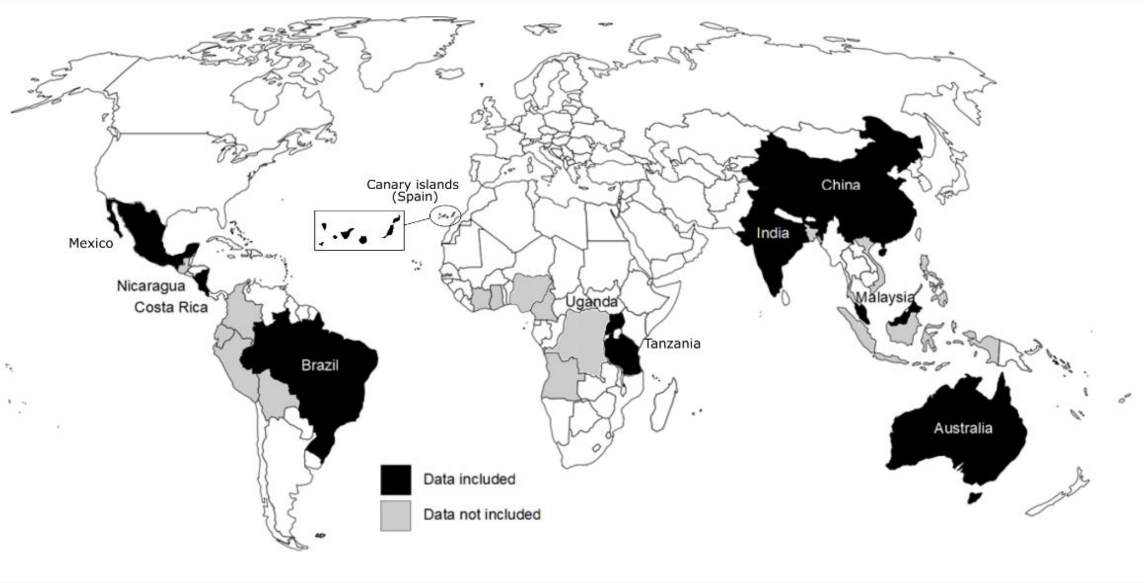

Fig. S1 A map showing, in black, countries included in the meta-analysis of the bacterial microbiome of *Musa* spp. Countries in grey are major producers (FAOSTAT, 2022) for which data were not available and were not included in meta-analysis.

Table S4 The impact of soil, genotype and plant compartment on alpha diversity metrics assessed by ANOVA from the bacterial microbiome of *Musa* spp. These results derive from our pot experiment which included five distinct soils, three *Musa* spp. genotypes, and eight compartments, each with 10 replicates.

**Response variable**	**Predictor variable**	***F* value**	***P* value**
Predicted OTUs (Chao 1)	Compartment	272.0	<0.001 ***
	Soil	1.7	0.151
	Compartment: Soil	3.4	<0.001 ***
Shannon's Diversity Index	Compartment	154.0	<0.001 ***
	Soil	1.2	0.290
	Compartment: Soil	2.9	<0.001 ***
Phylogenetic Diversity (Faith's)	Compartment	242.8	<0.001 ***
	Soil	1.5	0.193
	Compartment: Soil	4.3	<0.001 ***
Predicted OTUs (Chao 1)	Compartment	118.1	<0.001 ***
	Genotype	0.4	0.695
	Compartment: Genotype	0.7	0.728
Shannon's Diversity Index	Compartment	74.3	<0.001 ***
	Genotype	0.3	0.747
	Compartment: Genotype	0.9	0.593
Phylogenetic Diversity (Faith's)	Compartment	109.9	<0.001 ***
	Genotype	0.6	0.562
	Compartment: Genotype	0.6	0.850

Table S5 The impact of soil, genotype and plant compartment on bacterial community composition as represented by Weighted UniFrac distances using PERMANOVA. These results derive from our pot experiment which included five distinct soils, three *Musa* spp. genotypes, and eight compartments, each with 10 replicates.

Response variable	Predictor variable	*F* value	R^2 (%)	*P* value
Community composition (Weighted unifrac)	Compartment	82.9	56.8	<0.001 ***
	Soil	5.5	2.2	<0.001 ***
	Compartment: Soil	2.4	6.4	<0.001 ***
Community composition (Weighted unifrac)	Compartment	35.8	51.9	<0.001 ***
	Genotype	0.3	0.1	0.799
	Compartment: Genotype	1.6	4.3	0.064

Table S6 Average percentage similarity plus/minus the standard deviation of various *Musa* spp. plant compartments. Percentages were produced using a Bayesian approach implemented through SourceTracker.

	Source							
**Sink**	**BS**	**AER**	**AEnR**	**BER**	**BEnR**	**R**	**PS**	**L**
**BS**	-	84.6 ± 5	67.3 ± 11	87.9 ± 3	77.1 ± 6	38.6 ± 11	25.6 ± 17	0.9 ± 2
**AER**	81.4 ± 10	-	76.4 ± 10	90.7 ± 4	82.1 ± 7	59.5 ± 12	37.7 ± 20	21.2 ± 15
**AEnR**	47.6 ± 34	78.1 ± 25	-	73.0 ± 26	87.4 ± 14	81.2 ± 16	66.2 ± 28	40.3 ± 30
**BER**	89.5 ± 3	90.4 ± 4	69.4 ± 12	-	81.7 ± 7	46.1 ± 12	27.7 ± 20	10.6 ± 12
**BEnR**	54.0 ± 31	81.8 ± 19	85.2 ± 13	80.9 ± 19	-	76.7 ± 19	60.7 ± 33	37.0 ± 35
**R**	36.0 ± 30	81.9 ± 17	84.8 ± 16	75.7 ± 22	82.8 ± 18	-	79.0 ± 19	62.9 ± 22
**PS**	54.9 ± 38	70.7 ± 31	77.1 ± 29	67.5 ± 33	75.0 ± 30	82.3 ± 28	-	63.2 ± 33
**L**	6.5 ± 20	74.2 ± 23	75.5 ± 30	60.3 ± 27	70.5 ± 28	95.9 ± 5	95.8 ± 4	-

Table S7 The influence of soil on the alpha diversity of bacterial communities within each compartment, as assessed by ANOVA. The results are for the *Musa* (AAA Group, Cavendish Subgroup) 'Williams' plants grown in five distinct soils in our pot experiment.

Response variable	Compartment	*F* value	*P* value	
Chao1	Bulk soil	2.4	0.062	
	Apical ectorhizosphere	3.1	0.024	*
	Apical endorhizosphere	5.5	0.001	**
	Basal ectorhizosphere	5.3	0.001	**
	Basal endorhizosphere	1.6	0.200	
	Rhizome	0.8	0.519	
	Pseudostem	3.1	0.026	*
	Leaf	1.5	0.210	
Shannon	Bulk soil	6.1	<0.001	***
	Apical ectorhizosphere	2.4	0.060	
	Apical endorhizosphere	5.1	0.002	**
	Basal ectorhizosphere	10.4	<0.001	***
	Basal endorhizosphere	2.9	0.013	*
	Rhizome	1.2	0.308	
	Pseudostem	1.4	0.237	
	Leaf	0.4	0.829	
Phylogenetic diversity (Faith's)	Bulk soil	4.1	0.007	**
	Apical ectorhizosphere	3.7	0.011	*
	Apical endorhizosphere	7.7	<0.001	***
	Basal ectorhizosphere	3.9	0.009	**
	Basal endorhizosphere	3.6	0.012	*
	Rhizome	0.5	0.714	
	Pseudostem	3.0	0.027	*
	Leaf	1.3	0.283	

Table S8 The influence of soil on the composition of bacterial communities, as represented by Weighted UniFrac distances, within compartments as assessed using PERMANOVA. The results are for the *Musa* (AAA Group, Cavendish Subgroup) 'Williams' plants grown in five distinct soils in our pot experiment.

**Compartment**	***F* value**	**R^2 (%)**	***P* value**	
Bulk soil	5.2	31.4	<0.001	***
Apical ectorhizosphere	1.5	11.7	0.193	
Apical endorhizosphere	4.2	28.5	0.005	**
Basal ectorhizosphere	7.6	40.3	<0.001	***
Basal endorhizosphere	4.0	26.3	0.004	**
Rhizome	3.7	24.5	0.012	*
Pseudostem	1.2	9.9	0.318	
Leaf	0.6	5.0	0.679	

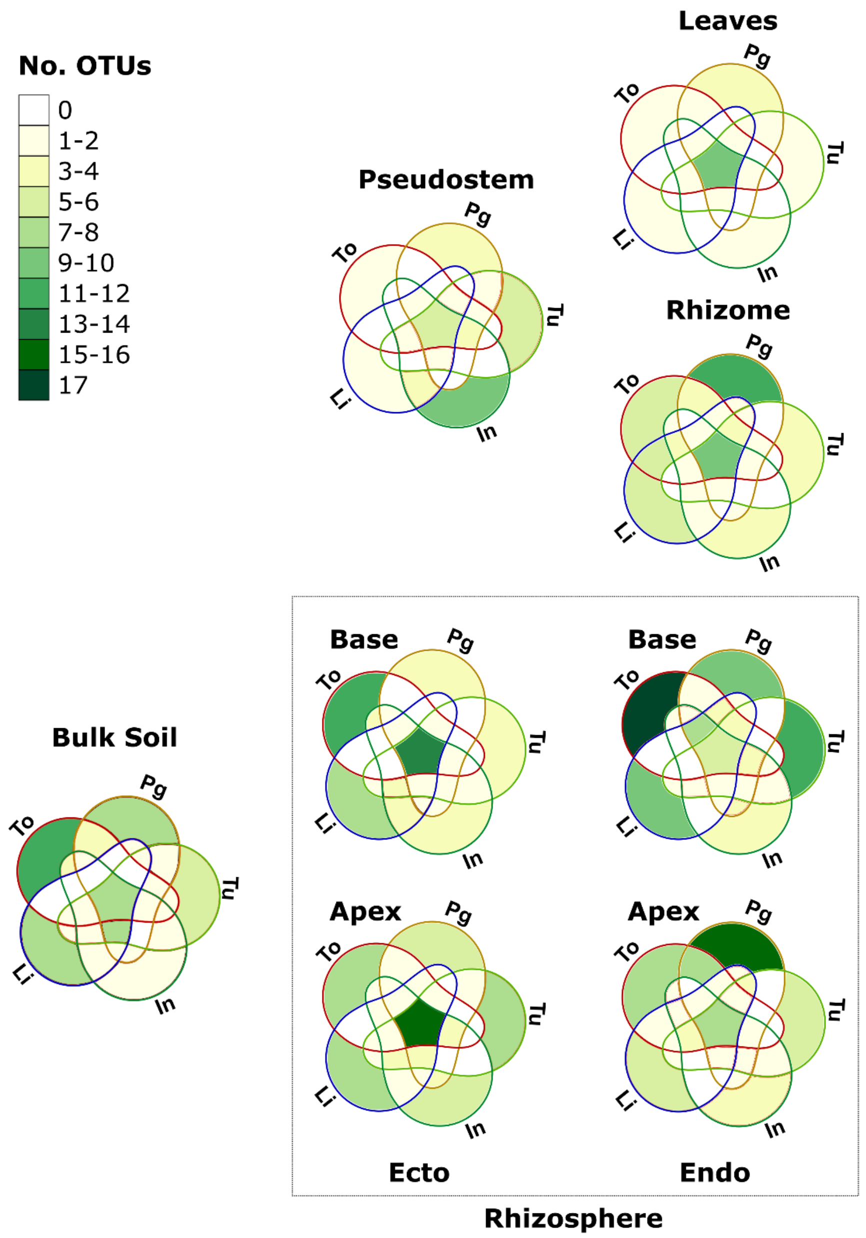

Fig. S2 Venn diagrams to show the numbers of shared candidate-core OTUs between *Musa* (AAA Group, Cavendish Subgroup) 'Williams' grown in five distinct soils (Pg, Tu, In, Li, and To) within compartments. Candidate-core OTUs are those that were present in ≥50% of the ten replicates within each treatment combination at a mean relative abundance of ≥0.5%. Core OTUs are those that were shared between all soils within each compartment. These results are from our pot experiment.

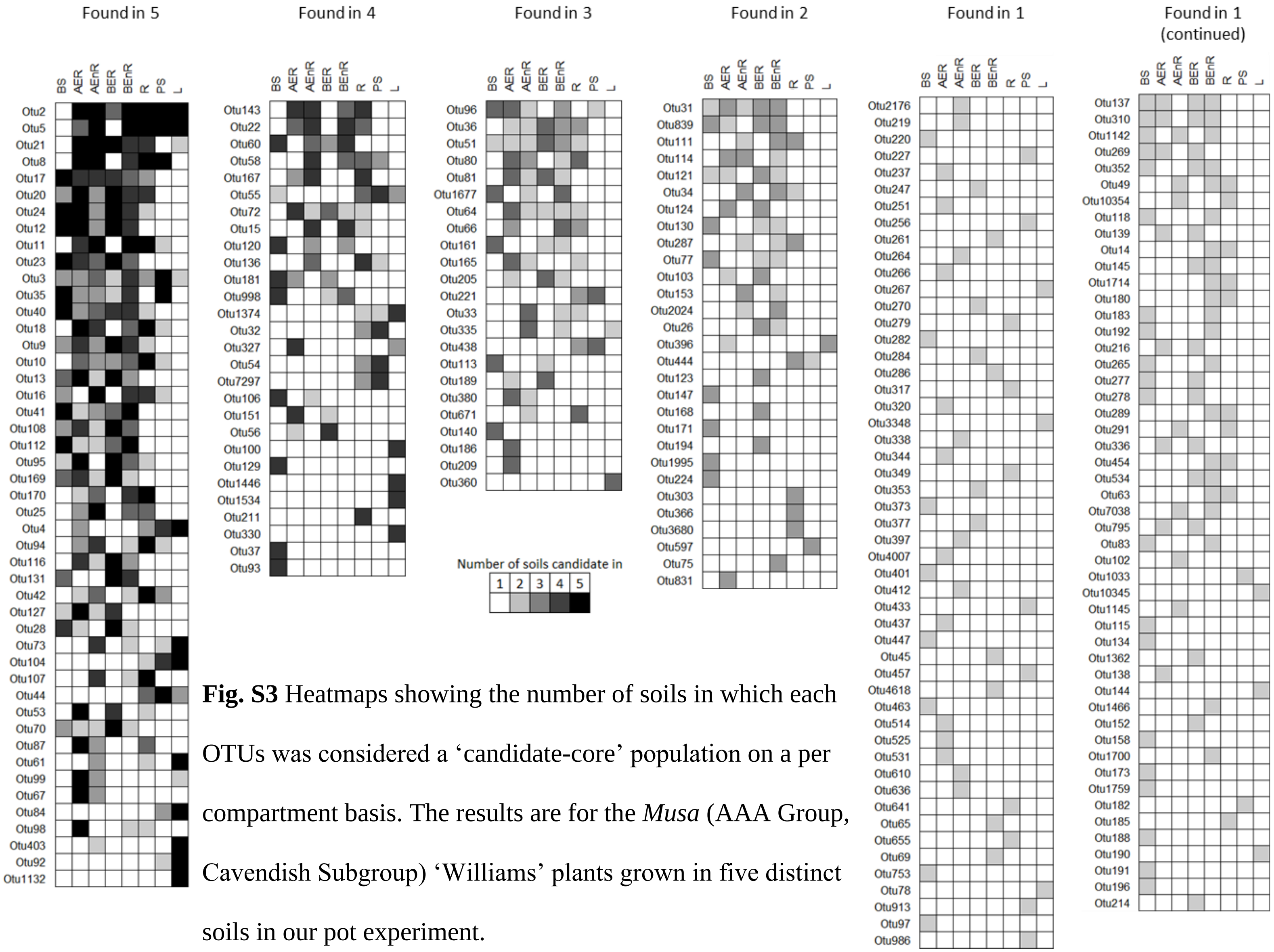

Fig. S3 Heatmaps showing the number of soils in which each OTUs was considered a ‘candidate-core’ population on a per compartment basis. The results are for the *Musa* (AAA Group, Cavendish Subgroup) ‘Williams’ plants grown in five distinct soils in our pot experiment.

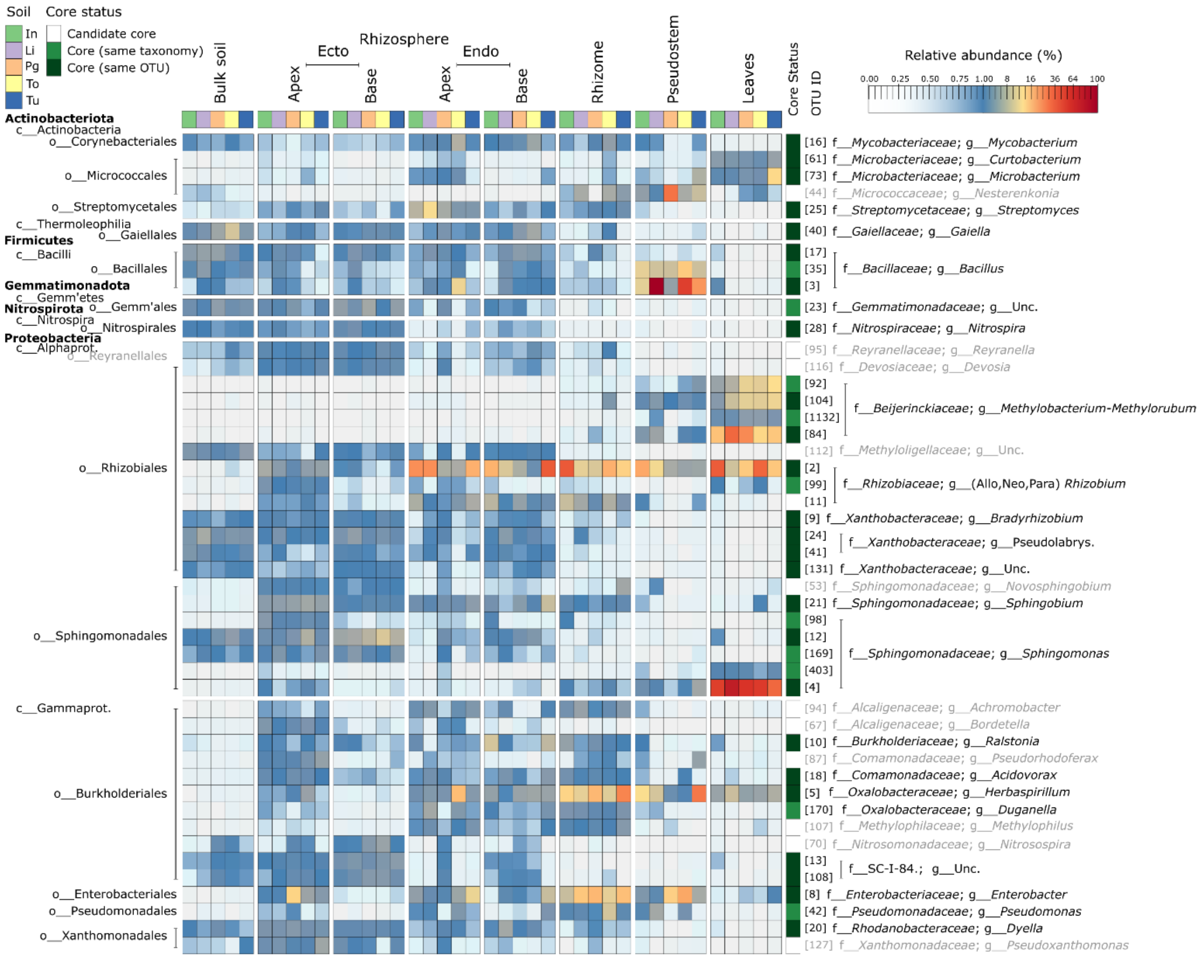

Fig. S4 The 'candidate' core bacterial microbiome of *Musa* spp. The heatmap highlights the relative abundances of core OTUs in each compartment and soil for the *Musa* (AAA Group, Cavendish Subgroup) 'Williams' plants within the pot experiment. Each cell represents the mean of the replicates for that treatment. OTUs in grey indicate 'candidates' that were not found in field-grown plants and were subsequently dropped. Those with black text were found in field plants and were elevated to full core status.

Table S9 OTUs found as candidate-core associated with *Musa* (AAA Group, Cavendish Subgroup) 'Williams' in plants grown in one to five soils or as key constituents in the field-grown *Musa* spp.

	**Total**	**OTUs**
Considered as candidate core in 5 soils	47	Otu2, Otu5, Otu21, Otu8, Otu17, Otu20, Otu24, Otu12, Otu11, Otu23, Otu3, Otu35, Otu40, Otu18, Otu9, Otu10, Otu13, Otu16, Otu41, Otu108, Otu112, Otu95, Otu169, Otu170, Otu25, Otu4, Otu94, Otu116, Otu131, Otu42, Otu127, Otu28, Otu73, Otu104, Otu107, Otu44, Otu53, Otu70, Otu87, Otu61, Otu99, Otu67, Otu84, Otu98, Otu403, Otu92, Otu1132
Considered as candidate core in 4 soils	28	Otu143, Otu22, Otu60, Otu58, Otu167, Otu55 Otu72, Otu15, Otu120, Otu136, Otu181, Otu998, Otu1374, Otu32, Otu327, Otu54, Otu7297, Otu106, Otu151, Otu56, Otu100, Otu129, Otu1446, Otu1534, Otu211, Otu330, Otu37, Otu93
Considered as candidate core in 3 soils	23	Otu96, Otu36, Otu51, Otu80, Otu81, Otu1677, Otu64, Otu66, Otu161, Otu165, Otu205, Otu221, Otu33, Otu335, Otu438, Otu113, Otu189, Otu380, Otu671, Otu140, Otu186, Otu209, Otu360
Considered as candidate core in 2 soils	29	Otu31, Otu839, Otu111, Otu114, Otu121, Otu34, Otu124, Otu130, Otu287, Otu77, Otu103, Otu153, Otu2024, Otu26, Otu396, Otu444, Otu123, Otu147, Otu168, Otu171, Otu194, Otu1995, Otu224, Otu303, Otu366, Otu3680, Otu597, Otu75, Otu831
Considered as candidate core in 1 soil	101	Otu137, Otu310, Otu1142, Otu269, Otu352, Otu49, Otu10354, Otu118, Otu139, Otu14, Otu145, Otu1714, Otu180, Otu183, Otu192, Otu216, Otu265, Otu277, Otu278, Otu289, Otu291, Otu336, Otu454, Otu534, Otu63, Otu7038, Otu795, Otu83, Otu102, Otu1033, Otu10345, Otu1145, Otu115, Otu134, Otu1362, Otu138, Otu144, Otu1466, Otu152, Otu158, Otu1700, Otu173, Otu1759, Otu182, Otu185, Otu188, Otu190, Otu191, Otu196, Otu214, Otu2176, Otu219, Otu220, Otu227, Otu237, Otu247, Otu251, Otu256, Otu261, Otu264, Otu266, Otu267, Otu270, Otu279, Otu282, Otu284, Otu286, Otu317, Otu320, Otu3348, Otu338, Otu344, Otu349, Otu353, Otu373, Otu377, Otu397, Otu4007, Otu401, Otu412, Otu433, Otu437, Otu447, Otu45, Otu457, Otu4618, Otu463, Otu514, Otu525, Otu531, Otu610, Otu636, Otu641, Otu65, Otu655, Otu69, Otu753, Otu78, Otu913, Otu97, Otu986
Key field constituents	90	Otu129, Otu101, Otu9, Otu40, Otu195, Otu68, Otu146, Otu56, Otu77, Otu130, Otu37, Otu38, Otu27, Otu24, Otu191, Otu118, Otu23, Otu13, Otu26, Otu57, Otu82, Otu62, Otu1677, Otu93, Otu86, Otu41, Otu131, Otu134, Otu47, Otu17, Otu75, Otu60, Otu29, Otu12, Otu83, Otu43, Otu88, Otu839, Otu108, Otu10, Otu46, Otu36, Otu30, Otu76, Otu72, Otu50, Otu28, Otu128, Otu3, Otu21, Otu133, Otu103, Otu20, Otu65, Otu164, Otu48, Otu2, Otu25, Otu18, Otu8, Otu34, Otu14, Otu5, Otu11, Otu1194, Otu16, Otu203, Otu135, Otu3680, Otu59, Otu73, Otu4, Otu6, Otu156, Otu218, Otu100, Otu335, Otu63, Otu52, Otu104, Otu229, Otu1333, Otu61, Otu1614, Otu239, Otu84, Otu446, Otu78, Otu436, Otu324

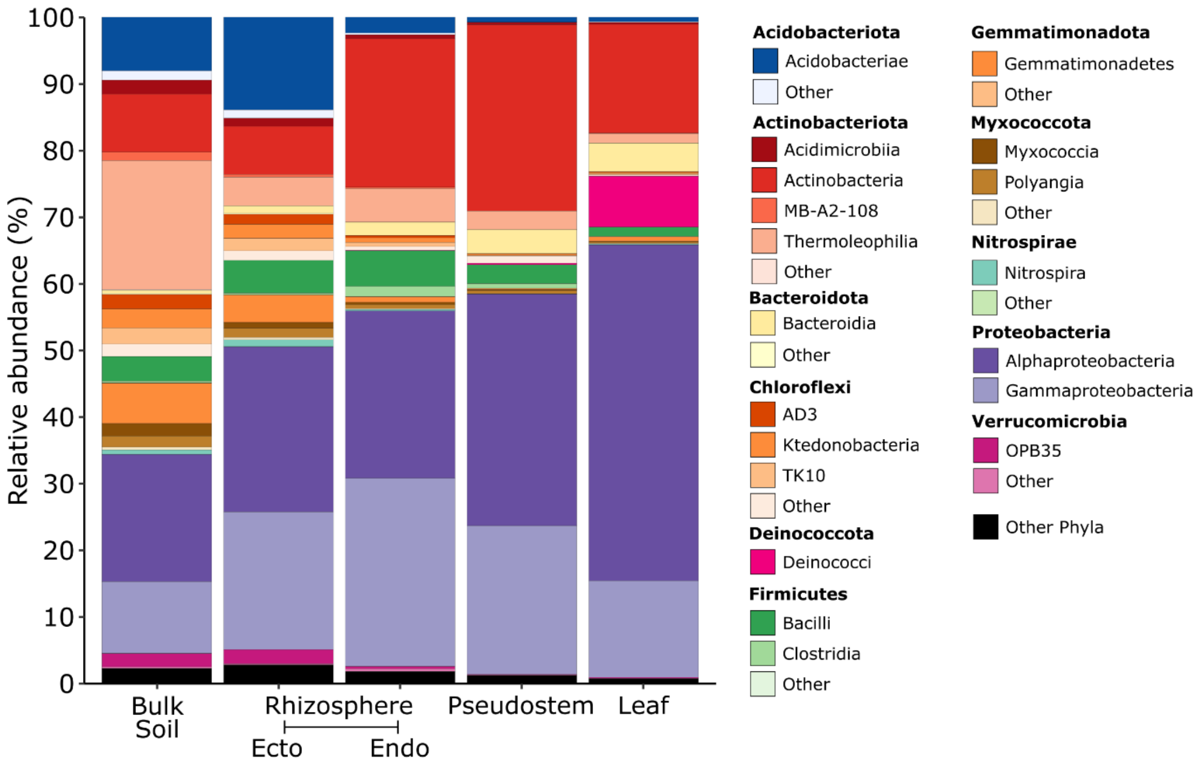

Fig. S5 The mean relative frequencies of bacterial classes associated with each compartment in field-grown *Musa* spp. Within each phylum, classes represented at <1% mean relative abundance are grouped as other.

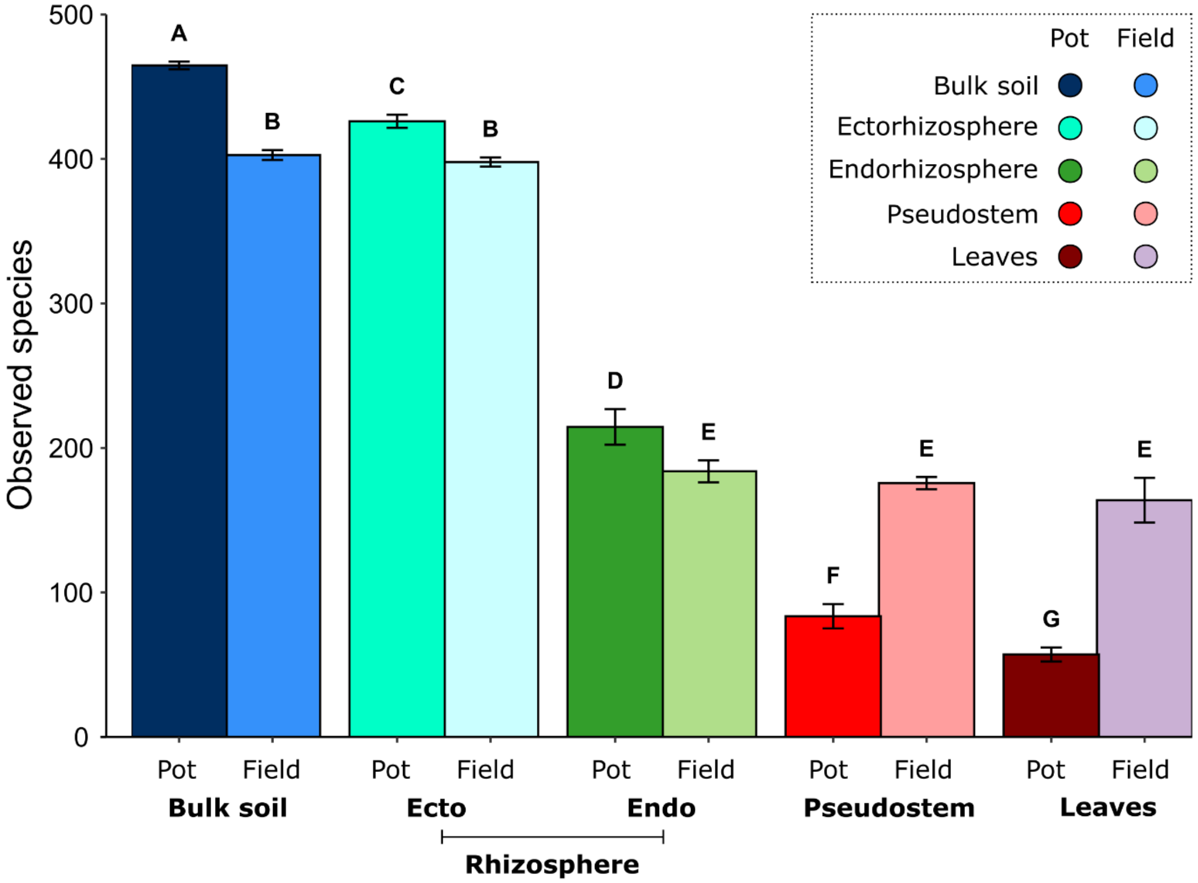

Fig. S6 Numbers of observed bacterial OTUs associated with different *Musa* spp. compartments and settings (pot vs. field). Error bars represent standard errors of the means. The letters indicate treatments that differ across soils according to estimated marginal means *post hoc* tests with Bejamini-Hochberg corrections. Members of the same groupings share the same letter.

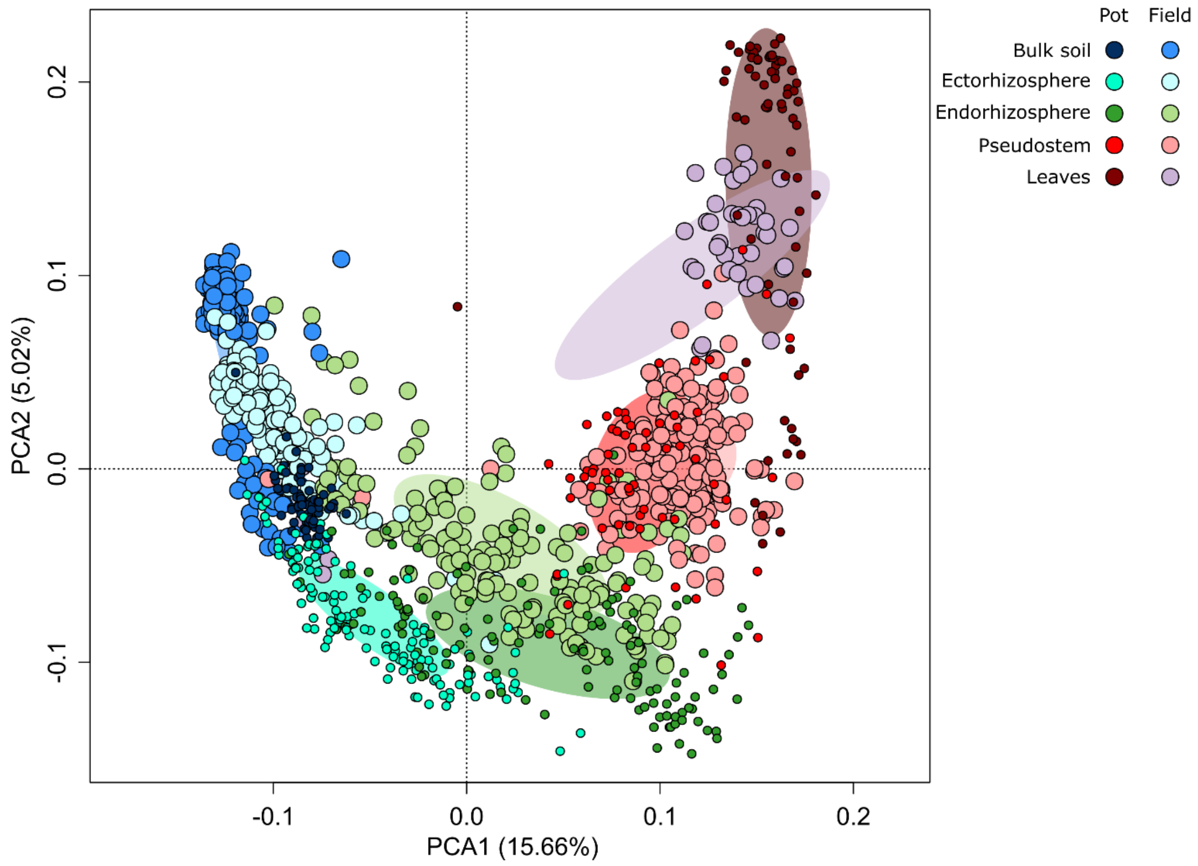

Fig. S7 A Principal Coordinate Analysis (PCA) ordination highlighting differences in the composition of bacterial communities (Hellinger transformed OTUs) associated with pot and field-grown *Musa* spp. in different plant compartments. Points representing field samples are larger than those representing pot samples. The ellipses represent standard deviations of the group centroids.

Table S10 SPIEC-EASI network centrality metrics for core OTUs in field-grown *Musa* spp.

OTU	Status	Taxonomy	Degree	Weighted degree	Betweenness	Closeness	Markov	PageRank
Otu12	Core	g__Sphingomonas	77	6.765	23148	4.36E-05	6.15E-04	1.58E-02
Otu13	Core	o__SC-I-84; f__; g__	43	3.347	10863	4.35E-05	6.02E-04	1.05E-02
Otu17	Core	g__Bacillus	39	2.714	5218	4.33E-05	5.98E-04	9.42E-03
Otu73	Core	g__Microbacterium	33	2.613	5390	4.32E-05	5.93E-04	8.35E-03
Otu41	Core	g__Pseudolabrys	27	2.380	4489	4.32E-05	5.81E-04	1.03E-02
Otu108	Core	o__SC-I-84;f__;g__	23	2.316	4777	4.33E-05	5.81E-04	8.39E-03
Otu28	Core	g__Nitrospira	22	1.360	1903	4.33E-05	5.80E-04	5.16E-03
Otu2	Core	g__(Allo,Neo,Para) Rhizobium	21	1.323	1869	4.32E-05	5.73E-04	6.39E-03
Otu24	Core	g__Pseudolabrys	21	1.315	1159	4.33E-05	5.77E-04	5.28E-03
Otu23	Core	f__Gemmatimonadaceae; g__	19	0.896	2440	4.32E-05	5.69E-04	4.45E-03
Otu40	Core	g__Gaiella	18	1.448	1571	4.32E-05	5.62E-04	6.82E-03
Otu61	Core	g__Curtobacterium	17	1.602	470	4.28E-05	5.56E-04	5.46E-03
Otu131	Core	f__Xanthobacteraceae; g__	15	1.167	1233	4.32E-05	5.55E-04	4.79E-03
Otu104	Core	g__Methylobacterium-Methylorubum	11	0.720	210	4.28E-05	5.27E-04	2.55E-03
Otu25	Core	g__Streptomyces	10	1.250	1648	4.27E-05	4.93E-04	6.33E-03
Otu4	Core	g__Sphingomonas	9	0.911	193	4.28E-05	5.08E-04	3.42E-03
Otu5	Core	g__Acidovorax	9	0.327	1572	4.32E-05	5.15E-04	1.15E-03
Otu9	Core	g__Bradyrhizobium	9	0.676	2153	4.30E-05	5.08E-04	2.76E-03
Otu11	Core	g__ (Allo,Neo,Para) Rhizobium	9	0.470	1338	4.28E-05	4.83E-04	3.34E-03
Otu21	Core	g__Sphingobium	8	0.380	144	4.30E-05	5.13E-04	1.83E-03
Otu84	Core	g__Methylobacterium-Methylorubum	8	1.279	129	4.26E-05	4.92E-04	4.46E-03
Otu403	Core	g__Sphingomonas	8	0.742	411	4.28E-05	5.04E-04	3.04E-03
Otu18	Core	g__Herbaspirillum	7	0.328	767	4.31E-05	4.93E-04	1.36E-03
Otu20	Core	g__Dyella	7	0.488	451	4.25E-05	4.51E-04	3.39E-03
Otu98	Core	g__Sphingomonas	6	0.569	9	4.27E-05	4.64E-04	2.73E-03
Otu99	Core	g__(Allo,Neo,Para) Rhizobium	6	0.234	110	4.28E-05	4.73E-04	1.38E-03
Otu170	Core	g__Duganella	6	0.255	937	4.29E-05	4.56E-04	2.95E-03
Otu8	Core	g__Enterobacter	5	0.590	200	4.27E-05	4.40E-04	4.03E-03
Otu10	Core	g__Ralstonia	5	0.409	296	4.30E-05	4.56E-04	1.90E-03
Otu42	Core	g__Pseudomonas	5	0.196	49	4.30E-05	4.59E-04	8.33E-04
Otu92	Core	g__Methylobacterium-Methylorubum	5	0.424	202	4.27E-05	4.52E-04	1.78E-03
Otu169	Core	g__Sphingomonas	5	0.564	310	4.32E-05	4.64E-04	2.37E-03
Otu16	Core	g__Mycobacterium	4	0.609	969	4.26E-05	4.18E-04	3.11E-03
Otu3	Core	g__Bacillus	3	0.573	0	4.28E-05	3.33E-04	2.78E-03
Otu35	Core	g__Bacillus	2	0.213	0	4.26E-05	3.00E-04	1.25E-03
Otu1132	Core	g__Methylobacterium-Methylorubum	0	-	-	-	-	-
Otu87	Non-core	g__Pseudorhodoferax	12	0.445	307	4.29E-05	5.37E-04	2.25E-03
Otu70	Non-core	g__Nitrosospira	9	0.688	778	4.28E-05	4.99E-04	3.51E-03
Otu127	Non-core	g__Pseudoxanthomonas	7	0.322	48	4.29E-05	4.95E-04	1.56E-03
Otu95	Non-core	g__Reyranella	6	0.501	116	4.31E-05	4.78E-04	2.23E-03
Otu112	Non-core	f__Methyloligellaceae; g__	5	0.113	111	4.25E-05	4.33E-04	1.34E-03
Otu116	Non-core	g__Devosia	4	0.314	312	4.30E-05	4.33E-04	1.36E-03
Otu94	Non-core	g__Achromobacter	3	0.255	86	4.26E-05	3.68E-04	1.94E-03
Otu107	Non-core	g__Methylophilus	3	0.143	24	4.25E-05	3.76E-04	1.07E-03
Otu44	Non-core	g__Nesterenkonia	1	0.006	0	5.31E-06	7.43E-05	2.53E-03
Otu53	Non-core	g__Novosphingobium	0	0.000	0	5.30E-06	7.41E-05	3.80E-04
Otu67	Non-core	g__Bordetella	0	-	-	-	-	-
Core median			8	0.57	411	4.29E-05	4.93E-04	2.95E-03
Non-core median			5	0.29	49	4.27E-05	4.37E-04	2.71E-03

*Otu67 & Otu1132 were not included in the field network as they did not meet the abundance / prevalence threshold

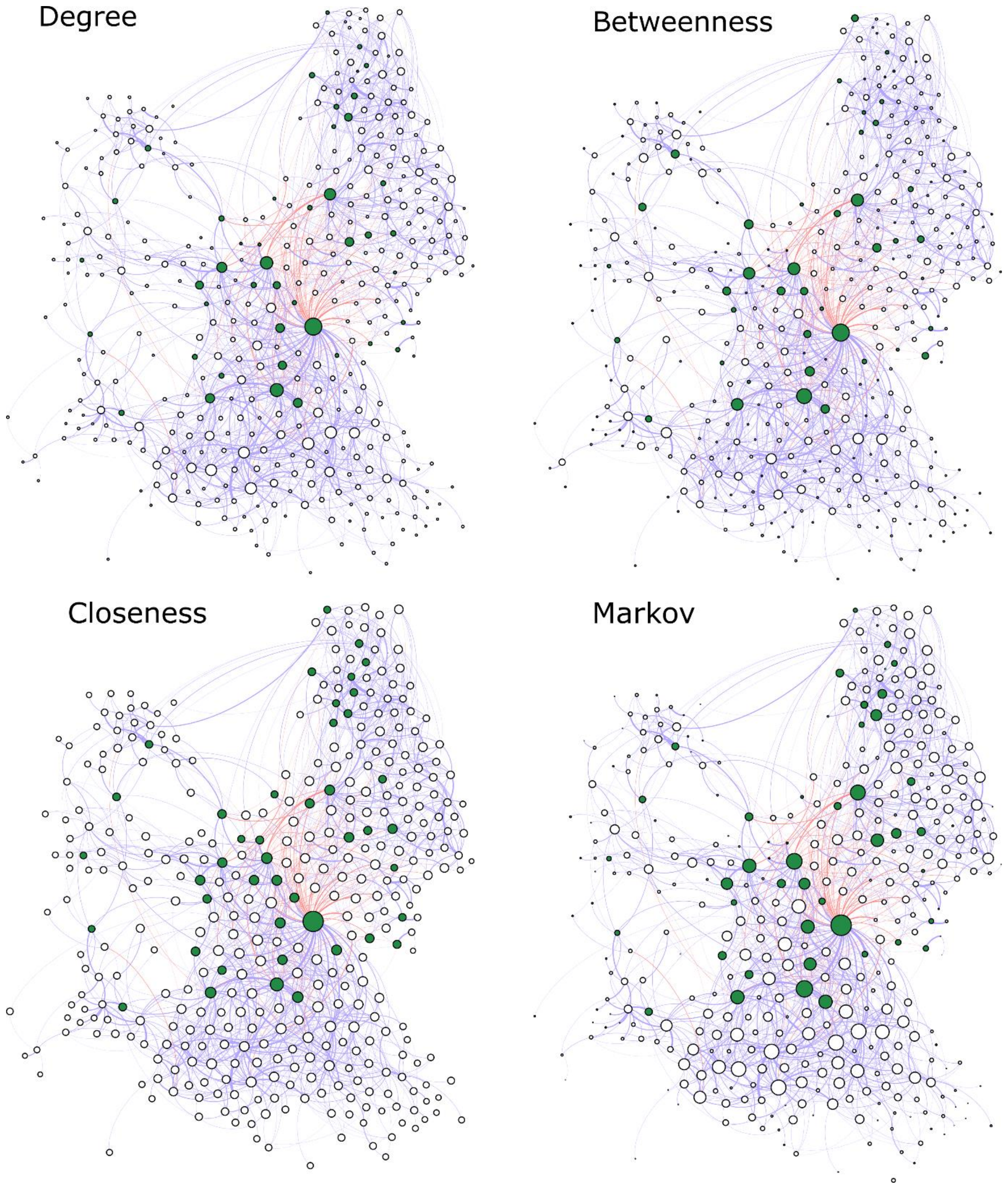

Fig. S8 SPIEC-EASI network graphs showing the co-occurrences of core and non-core bacteria in field-grown *Musa* spp. Nodes are coloured by core status and size is positively associated with a range of centrality metrics. Edge colours represent positive (blue) and negative (red) associations between taxa. Edge width is positively associated with the coefficient for the co-occurrence between the taxa. The node layout in each graph is the same as in Figure 4, which shows the OTU IDs.

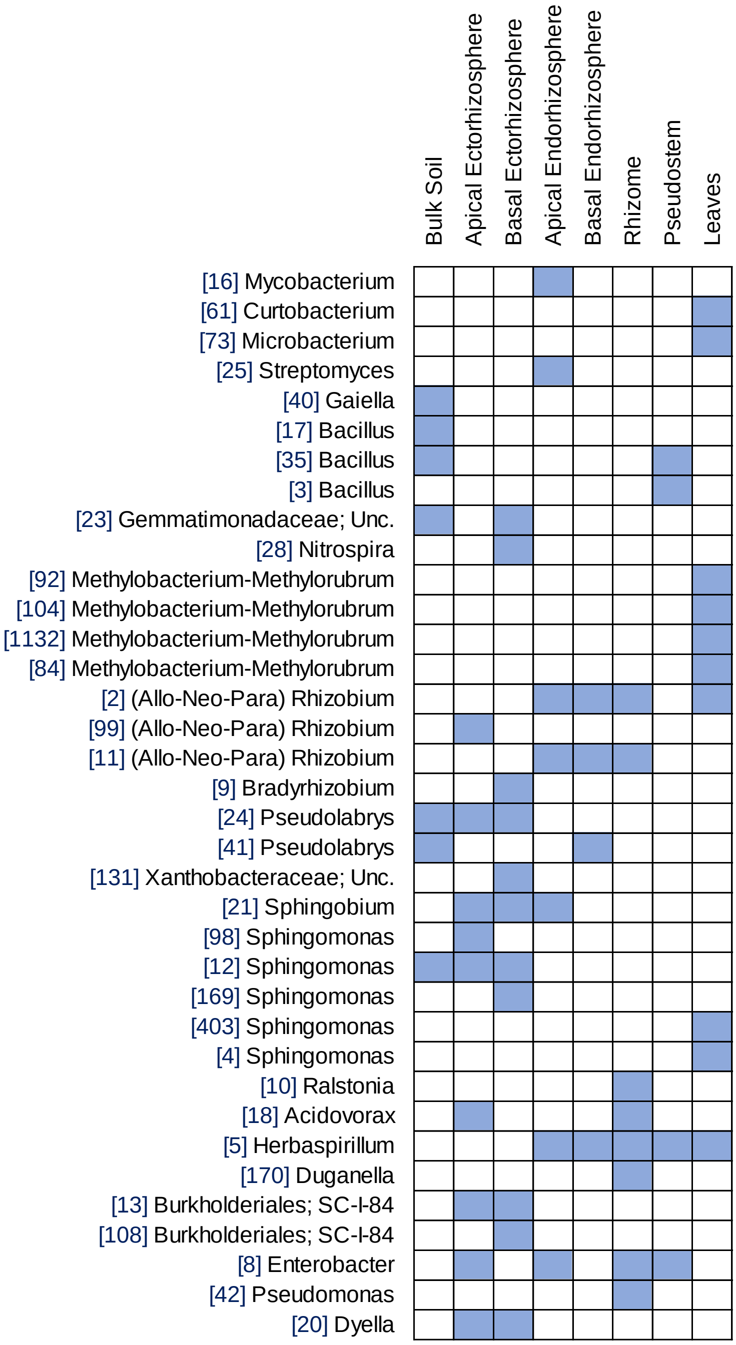

Fig. S9 The common core bacterial microbiome of *Musa* spp. Blue tiles highlight which OTUs are core within each plant compartment.

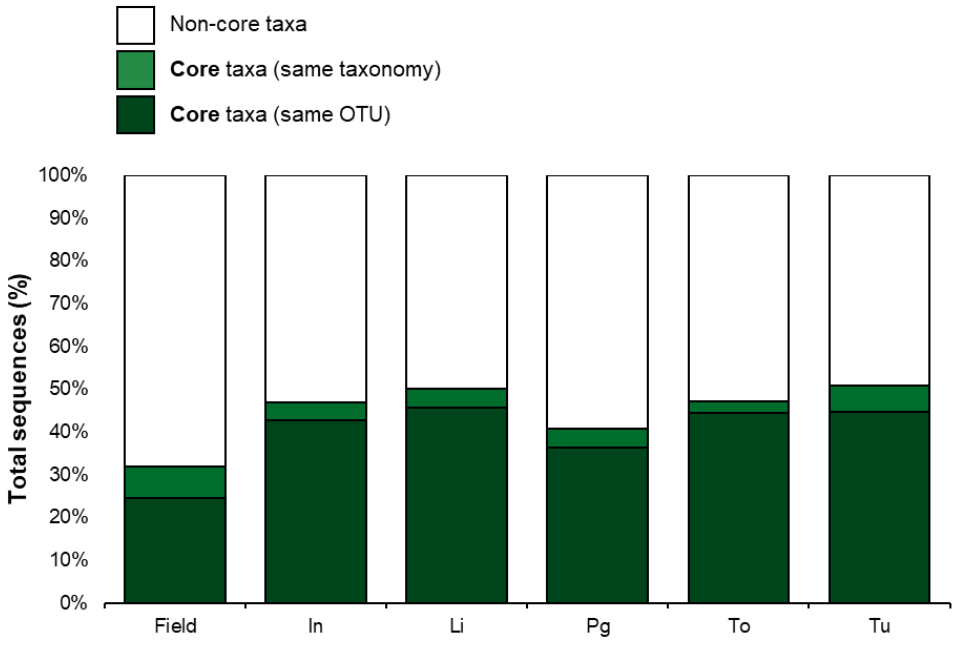

Fig. S10 Percentage of total bacterial sequences attributable to core OTUs and non-core OTUs within *Musa* spp. grown in the field or in pots containing different soils (In, Li, Pg, To, Tu).

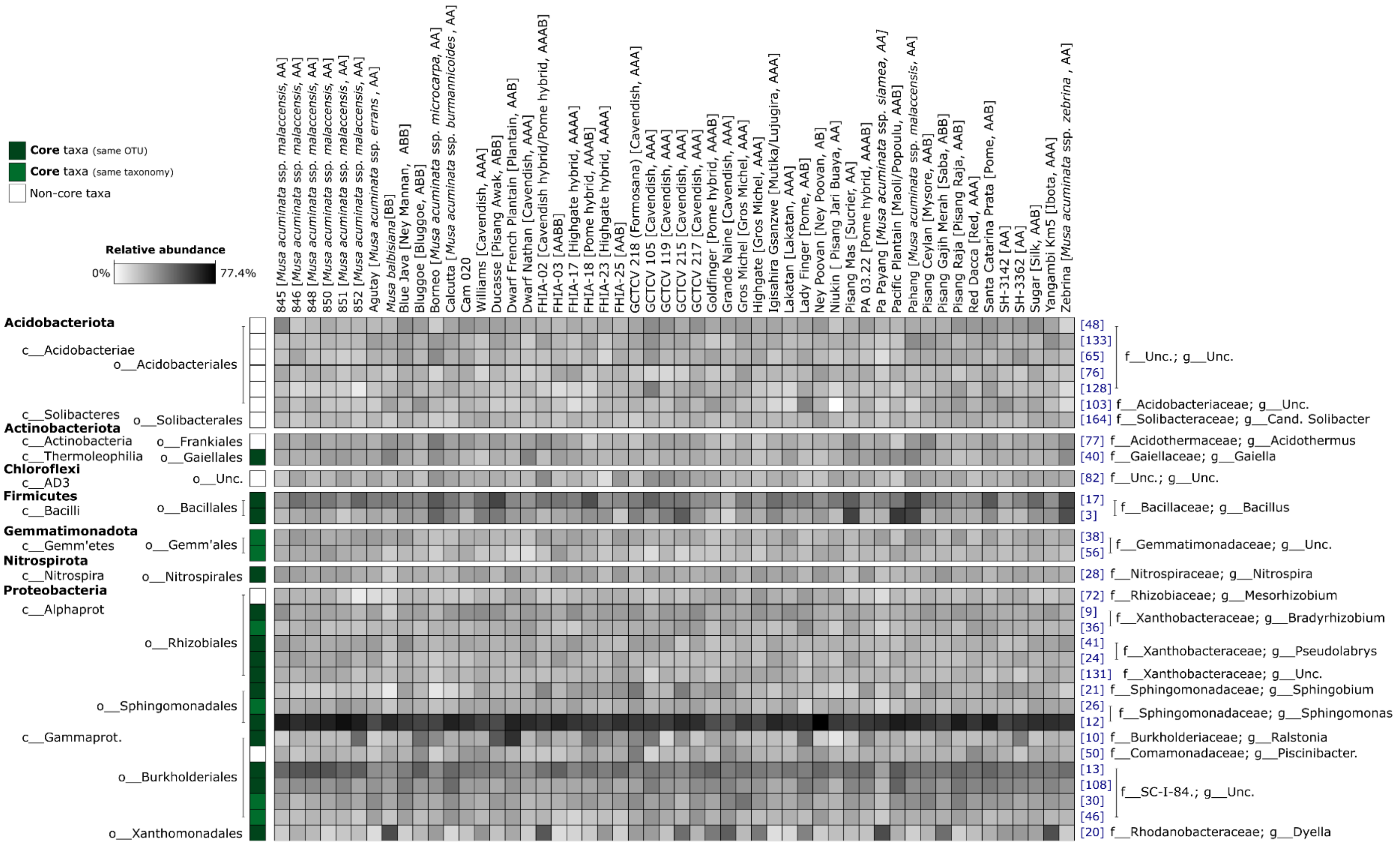

Fig. S11 A heatmap representing the mean relative abundances of key constituent OTUs in the ectorhizosphere of field-grown *Musa* spp. (i.e. present at ≥0.5% relative abundance in ≥50% of samples). The green squares indicate taxa that are members of the core microbiome.

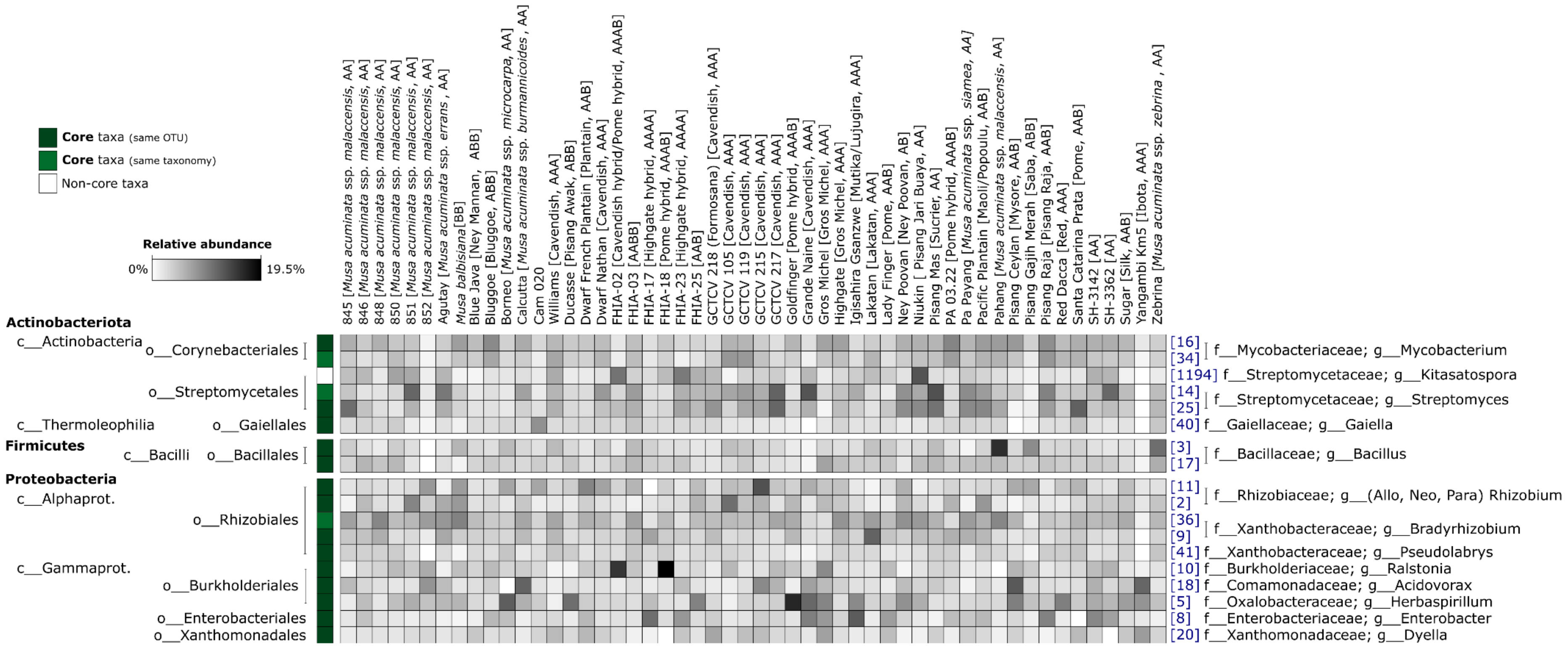

Fig. S12 A heatmap representing the mean relative abundances of key constituent OTUs in the endorhizosphere of field-grown *Musa* spp. (i.e. present at ≥0.5% relative abundance in ≥50% of samples). The green squares indicate taxa that are members of the core microbiome.

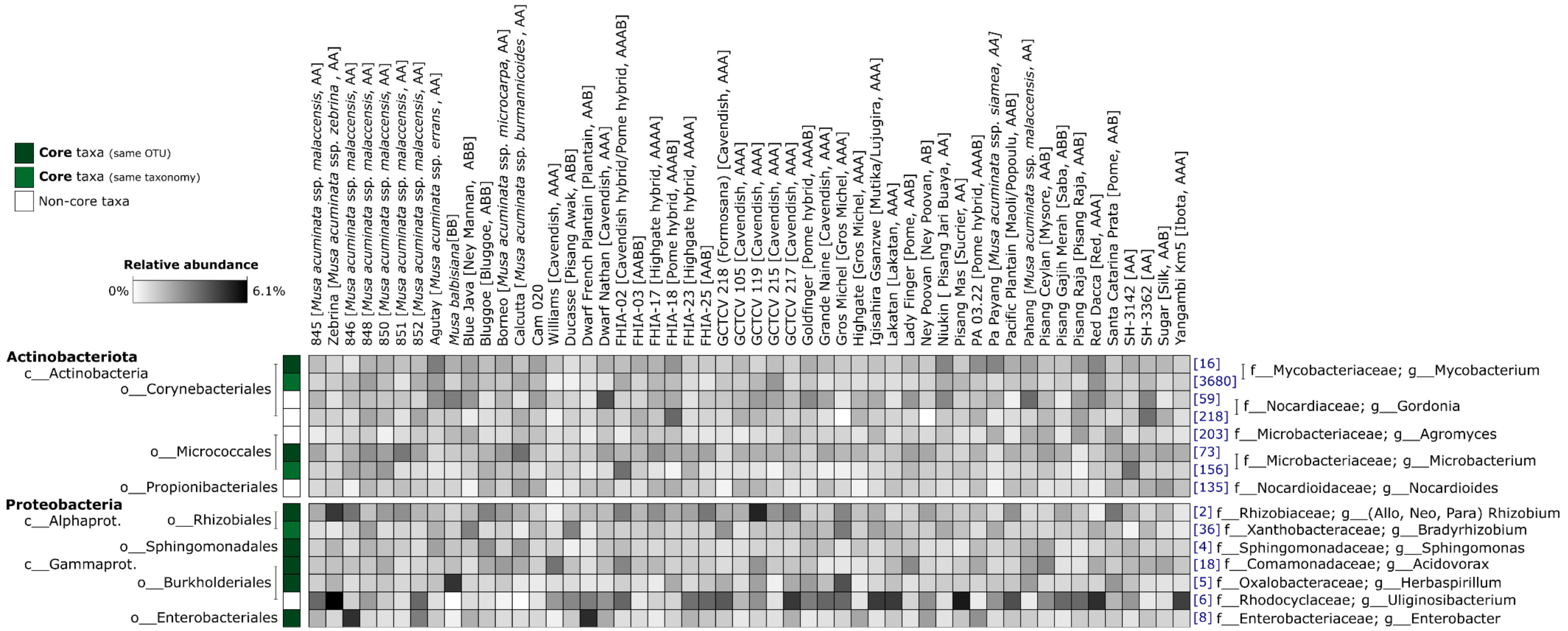

Fig. S13 A heatmap representing the mean relative abundances of key constituent OTUs in the pseudostem of field-grown *Musa* spp. (i.e. present at ≥0.5% relative abundance in ≥50% of samples). The green squares indicate taxa that are members of the core microbiome.

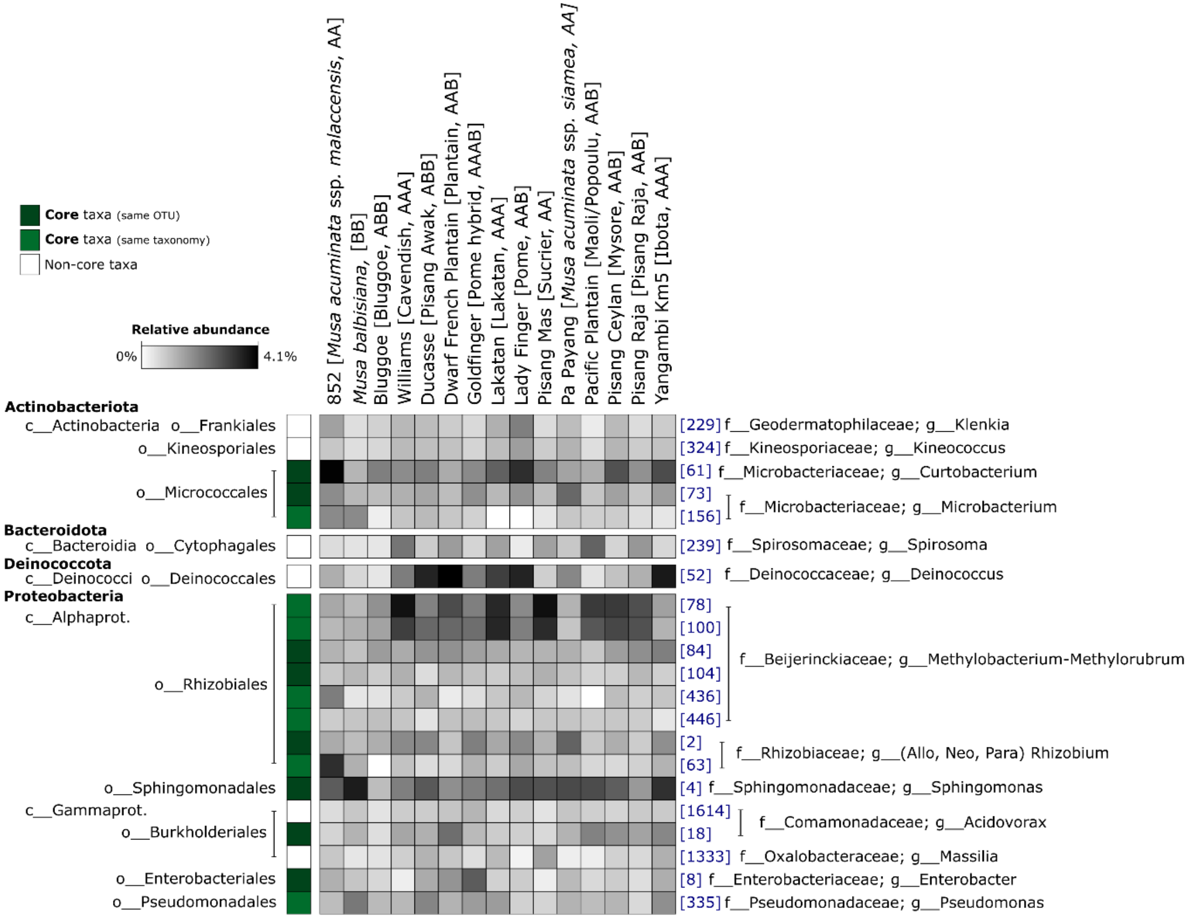

Fig. S14 A heatmap representing the mean relative abundances of key constituent OTUs in the leaves of field-grown *Musa* spp. (i.e. present at ≥0.5% relative abundance in ≥50% of samples). The green squares indicate taxa that are members of the core microbiome.

Table S11 Representative sequences the 47 candidate-core OTUs identified in the *Musa* (AAA Group, Cavendish Subgroup) 'Williams' plants grown in the five distinct soils in our pot experiment. OTUs that were elevated to full core-status are marked as core in bold text.

OTU	Sequence (5'-3')
**Otu2 Core**	GTAGTCCACGCCGTAAACGATGAATGTTAGCCGTCGGGCAGTATACTGTTCGGTGGCGCAGCTAACGCATTAAACATTCCGCCTGGGGAGTACGGTCGCAAGA TTAAAACTCAAAGGAATTGACGGGGGCCCGCACAAGCGGTGGAGCATGTGGTTTAATTCGAAGCAACGCGCAGAACCTTACCAGCTCTTGACATTCGGGGTAT GGGCATTGGAGACGATGTCCTTCAGTTAGGCTGGCCCCAGAACA
**Otu3 Core**	GTAGTCCACGCCGTAAACGATGAGTGCTAAGTGTTAGAGGGTTTCCGCCCTTTAGTGCTGCAGCTAACGCATTAAGCACTCCGCCTGGGGAGTACGGTCGCAA GACTGAAACTCAAAGGAATTGACGGGGGCCCGCACAAGCGGTGGAGCATGTGGTTTAATTCGAAGCAACGCGAAGAACCTTACCAGGTCTTGACATCCTCTGA CAACTCTAGAGATAGAGCGTTCCCCTTCGGGGGACAGAGTGACA
**Otu4 Core**	GTAGTCCACGCCGTAAACGATGATAACTAGCTGTCCGGGCACTTGGTGCTTGGGTGGCGCAGCTAACGCATTAAGTTATCCGCCTGGGGAGTACGGCCGCAAG GTTAAAACTCAAAGGAATTGACGGGGGCCTGCACAAGCGGTGGAGCATGTGGTTTAATTCGAAGCAACGCGCAGAACCTTACCAGCGTTTGACATGTCCGGAC GACTTCCAGAGATGGATTTCTTCCCTTCGGGGACTGGAACACAG
**Otu5 Core**	GTAGTCCACGCCCTAAACGATGTCTACTAGTTGTCGGGTCTTAATTGACTTGGTAACGCAGCTAACGCGTGAAGTAGACCGCCTGGGGAGTACGGTCGCAAGA TTAAAACTCAAAGGAATTGACGGGGACCCGCACAAGCGGTGGATGATGTGGATTAATTCGATGCAACGCGAAAAACCTTACCTACCCTTGACATGGATGGAAT CCTGAAGAGATTTGGGAGTGCTCGAAAGAGAACCATCACACAGG
**Otu8 Core**	GTAGTCCACGCCGTAAACGATGTCGACTTGGAGGTTGTGCCCTTGAGGCGTGGCTTCCGGAGCTAACGCGTTAAGTCGACCGCCTGGGGAGTACGGCCGCAAG GTTAAAACTCAAATGAATTGACGGGGGCCCGCACAAGCGGTGGAGCATGTGGTTTAATTCGATGCAACGCGAAGAACCTTACCTACTCTTGACATCCAGAGAA CTTAGCAGAGATGCTTTGGTGCCTTCGGGAACTCTGAGACAGGT
**Otu9 Core**	GTAGTCCACGCCGTAAACGATGAATGCCAGCCGTTAGTGGGTTTACTCACTAGTGGCGCAGCTAACGCTTTAAGCATTCCGCCTGGGGAGTACGGTCGCAAGA TTAAAACTCAAAGGAATTGACGGGGGCCCGCACAAGCGGTGGAGCATGTGGTTTAATTCGACGCAACGCGCAGAACCTTACCAGCCCTTGACATCCCGGTCGC GGACTCCAGAGACGGAGTTCTTCAGTTCGGCTGGACCGGAGACA
**Otu10 Core**	GTAGTCCACGCCCTAAACGATGTCAACTAGTTGTTGGGGATTCATTTCCTTAGTAACGTAGCTAACGCGTGAAGTTGACCGCCTGGGGAGTACGGTCGCAAGA TTAAAACTCAAAGGAATTGACGGGGACCCGCACAAGCGGTGGATGATGTGGATTAATTCGATGCAACGCGAAAAACCTTACCTACCCTTGACATGCCACTAAC GAAGCAGAGATGCATTAGGTGCTCGAAAGAGAAAGTGGACACAG

Otu11 Core GTAGTCCACGCCGTAAACGATGAATGTTAGCCGTCGGGCAGTATACTGTTCGGTGGCGCAGCTAACGCATTAAACATTCCGCCTGGGGAGTACGGTCGCAAGA
TTAAAACTCAAAGGAATTGACGGGGGCCCGCACAAGCGGTGGAGCATGTGGTTTAATTCGAAGCAACGCGCAGAACCTTACCAGCCCTTGACATCCTGTGTTA
CCTCTAGAGATAGGGGGTCCACTTCGGTGGCGCAGAGACAGGTG

Otu12 Core GTAGTCCACGCCGTAAACGATGATGACTAGCTGTCGGGGCGCTTAGCGTTCCGGTGGCGCAGCTAACGCGTTAAGTCATCCGCCTGGGGAGTACGGCCGCAAG
GTTAAAACTCAAAGAAATTGACGGGGGCCTGCACAAGCGGTGGAGCATGTGGTTTAATTCGAAGCAACGCGCAGAACCTTACCAGCGTTTGACATGCCAGGAC
GGTTTCCAGAGATGGATTCCTTCCCTTACGGGACCTGGACACAG

Otu13 Core GTAGTCCACGCCCTAAACGATGTCGACTGGGTGTTGGGGAAGCGATTTCCCAGTACCGTAGCTAACGCGTGAAGTCGACCGCCTGGGGAGTACGGCCGCAAGG
TTAAAACTCAAAGGAATTGACGGGGACCCGCACAAGCGGTGGATGATGTGGATTAATTCGATGCAACGCGAACAACCTTACCTACCCTTGACATGTGCCGAAG
CCCGGTGAGAGCCGGGGGTGCCCGAAAGGGAACGGCAACACAGG

Otu16 Core GTAGTCCACGCCGTAAACGGTGGGTACTAGGTGTGGGTTTCCTTCCTTGGGATCCGTGCCGTAGCTAACGCATTAAGTACCCCGCCTGGGGAGTACGGCCGCA
AGGCTAAAACTCAAAGGAATTGACGGGGGCCCGCACAAGCGGCGGAGCATGTGGATTAATTCGATGCAACGCGAAGAACCTTACCTGGGTTTGACATGCACAG
GACGCCGGCAGAGATGTCGGTTCCCTTGTGGCCTGTGTGCAGGT

Otu17 Core GTAGTCCACGCCGTAAACGATGAGTGCTAAGTGTTAGAGGGTTTCCGCCCTTTAGTGCTGCAGCTAACGCATTAAGCACTCCGCCTGGGGAGTACGGCCGCAA
GGCTGAAACTCAAAGGAATTGACGGGGGCCCGCACAAGCGGTGGAGCATGTGGTTTAATTCGAAGCAACGCGAAGAACCTTACCAGGTCTTGACATCCTCTGA
CACTCCTAGAGATAGGACTTTCCCCTTCGGGGGACAGAGTGACA

Otu18 Core GTAGTCCACGCCCTAAACGATGTCAACTGGTTGTTGGGTCTTCACTGACTCAGTAACGAAGCTAACGCGTGAAGTTGACCGCCTGGGGAGTACGGCCGCAAGG
TTGAAACTCAAAGGAATTGACGGGGACCCGCACAAGCGGTGGATGATGTGGTTTAATTCGATGCAACGCGAAAAACCTTACCCACCTTTGACATGTACGGAAT
TTGCCAGAGATGGCTTAGTGCTCGAAAGAGAGCCGTAACACAGG

Otu20 Core GTAGTCCACGCCCTAAACGATGCGAACTGGATGTTGGTCTCAACTCGGAGATCAGTGTCGAAGCTAACGCGTTAAGTTCGCCGCCTGGGGAGTACGGTCGCAA
GACTGAAACTCAAAGGAATTGACGGGGGCCCGCACAAGCGGTGGAGTATGTGGTTTAATTCGATGCAACGCGAAGAACCTTACCTGGCCTTGACATGTCTGGA
ATCCTGCAGAGATGCGGGAGTGCCTTCGGGAATCAGAACACAGG

Otu21 Core GTAGTCCACGCCGTAAACGATGATAACTAGCTGCTGGGGTGCATGGCATTTCAGTGGCGCAGCTAACGCATTAAGTTATCCGCCTGGGGAGTACGGTCGCAAG
ATTAAAACTCAAAGGAATTGACGGGGGCCTGCACAAGCGGTGGAGCATGTGGTTTAATTCGAAGCAACGCGCAGAACCTTACCAACGTTTGACATCCCTATCG
CGGATCGTGGAGACACTTTCCTTCAGTTCGGCTGGATAGGTGAC

Otu23 Core GTAGTCCACGCCGTAAACGATGGACACTAGGCGTCGGGGGGAGCGACCCTCCCGGTGCCGTCGCTAACGCAGTAAGTGTCCCGCCTGGGGAGTACGGCCGCAA
GGCTGAAACTCAAAGGAATTGACGGGGGCCCGCACAAGCGGTGGAGCATGTGGTTTAATTCGAAGCAACGCGAAGAACCTTACCTGGGCTTGACATGCACGGG
AAAGCCGGTGGAAACATCGGCCCCTCTTCGGAGTCCGTGCACAG

Otu24
Core

GTAGTCCACGCTGTAAACGATGGATGCTAGCCGTTGGCGAGCTTGCTCGTCAGTGGCGCAGCTAACGCTTTAAGCATCCCGCCTGGGGAGTACGGTCGCAAGA
TTAAAACTCAAAGGAATTGACGGGGGCCCGCACAAGCGGTGGAGCATGTGGTTCAATTCGAAGCAACGCGCAGAACCTTACCAGCCCTTGACATGTCCTCTAT
GGATTCCAGAGACGGAGTCCTTCACTTCGGGTGGAGGAACACA

Otu25
Core

GTAGTCCACGCCGTAAACGGTGGGAACTAGGTGTTGGCGACATTCCACGTCGTCGGTGCCGCAGCTAACGCATTAAGTTCCCCGCCTGGGGAGTACGGCCGCA
AGGCTAAAACTCAAAGGAATTGACGGGGGCCCGCACAAGCAGCGGAGCATGTGGCTTAATTCGACGCAACGCGAAGAACCTTACCAAGGCTTGACATACACCG
GAAACGGCCAGAGATGGTCGCCCCCTTGTGGTCGGTGTACAGGT

Otu28
Core

GTAGTCCACGCCTTAAACGATGGATACTAAGTGTCGGCGGGTTACCGCCGGTGCCGCAGCTAACGCAGTAAGTATCCCGCCTGGGAAGTACGGCCGCAAGGTT
GAAACTCAAAGGAATTGACGGGGGCCCGCACAAGCGGTGGAGCATGTGGTTTAATTCGACGCAACGCGAAGAACCTTACCTAGGCTGGACATGCACGTAGTAG
AAGGATGAAAGTCTAACGAGGTAGCAATACCAGCGTGCTCAGGT

Otu35
Core

GTAGTCCACGCCGTAAACGATGAGTGCTAAGTGTTAGGGGGTTTCCGCCCCTTAGTGCTGCAGCTAACGCATTAAGCACTCCGCCTGGGGAGTACGGTCGCAA
GACTGAAACTCAAAGGAATTGACGGGGGCCCGCACAAGCGGTGGAGCATGTGGTTTAATTCGAAGCAACGCGAAGAACCTTACCAGGTCTTGACATCCTCTGA
CAACCCTAGAGATAGGGCTTTCCCTTCGGGGACAGAGTGACAGG

Otu40
Core

GTAGTCCACGCCGTAAACGATGGGTGCTAGGTGTGGGCGGTGTCGACTCCGTCCGTGCCGAAGCTAACGCATTAAGCACCCCGCCTGGGGAGTACGGCCGCAA
GGCTAAAACTCAAAGGAATTGACGGGGGCCCGCACAAGCAGCGGAGCATGTTGTTTAATTCGACGCGACGCGAAGAACCTTACCAAGGCTTGACATGCACGGG
AATGTCGTAGAAATACGGCAGCCCTTCGGGGCTCGTGCACAGGT

Otu41
Core

GTAGTCCACGCCGTAAACGATGGATGCTAGCCGTTGGTGGGTTTACCCATCAGTGGCGCAGCTAACGCATTAAGCATCCCGCCTGGGGAGTACGGTCGCAAGA
TTAAAACTCAAAGGAATTGACGGGGGCCCGCACAAGCGGTGGAGCATGTGGTTCAATTCGAAGCAACGCGAAGAACCTTACCAGCCTTTGACATCCCGGTCGC
GAGCACCAGAGATGGAGCTCTTCAGTTCGGCTGGACCGGAGACA

Otu42
Core

GTAGTCCACGCCGTAAACGATGTCAACTAGCCGTTGGAATCCTTGAGATTTTAGTGGCGCAGCTAACGCATTAAGTTGACCGCCTGGGGAGTACGGCCGCAAG
GTTAAAACTCAAATGAATTGACGGGGGCCCGCACAAGCGGTGGAGCATGTGGTTTAATTCGAAGCAACGCGAAGAACCTTACCAGGCCTTGACATGCAGAGAA
CTTTCCAGAGATGGATTGGTGCCTTCGGGAACTCTGACACAGGT

Otu44

GTAGTCCATGCCGTAAACGTTGGGCACTAGGTGTGGGGGACATTCCACGTTTTCCGCGCCGTAGCTAACGCATTAAGTGCCCCGCCTGGGGAGTACGGCCGCA
AGGCTAAAACTCAAAGGAATTGACGGGGGCCCGCACAAGCGGCGGAGCATGCGGATTAATTCGATGCAACGCGAAGAACCTTACCAAGGCTTGACATGGACCG
GACCGCTGCAGAGATGTAGCTTCCCTTCGGGGCTGGTTCACAGG

Otu53

GTAGTCCACGCCGTAAACGATGATAACTAGCTGTCCGGGCACTTGGTGCTTGGGTGGCGCAGCTAACGCATTAAGTTATCCGCCTGGGGAGTACGGTCGCAAG
ATTAAAACTCAAAGGAATTGACGGGGGCCTGCACAAGCGGTGGAGCATGTGGTTTAATTCGAAGCAACGCGCAGAACCTTACCAGCCTTTGACATCCCGCGCT
ACTTCCAGAGATGGAAGGTTCCCTTCGGGGACGCGGTGACAGGT

Otu61 Core

GTAGTCCACGCCGTAAACGTTGGGCGCTAGATGTAGGGACCTTTCCACGGTTTCTGTGTCGTAGCTAACGCATTAAGCGCCCCGCCTGGGGAGTACGGCCGCA
AGGCTAAAACTCAAAGGAATTGACGGGGGCCCGCACAAGCGGCGGAGCATGCGGATTAATTCGATGCAACGCGAAGAACCTTACCAAGGCTTGACATACACCG
GAAACGGCCAGAGATGGTCGCCCCCTTGTGGTCGGTGTACAGGT

Otu67

GTAGTCCACGCCCTAAACGATGTCAACTAGCTGTTGGGGGCTTCGGCTCTTGGTAGCGCAGCTAACGCGTGAAGTTGACCGCCTGGGGAGTACGGTCGCAAGA
TTAAAACTCAAAGGAATTGACGGGGACCCGCACAAGCGGTGGATGATGTGGATTAATTCGATGCAACGCGAAAAACCTTACCTACCCTTGACATGTCTGGAAG
GCTAAAGAGATTTGGCTGTGCTCGCAAGAGAACTGGAACACAGG

Otu70

GTAGTCCACGCCCTAAACGATGTCAACTAGTTGTCGGGTCTTAACGGACTTGGTAACGCAGCTAACGCGTGAAGTTGACCGCCTGGGGAGTACGGTCGCAAGA
TTAAAACTCAAAGGAATTGACGGGGACCCGCACAAGCGGTGGATTATGTGGATTAATTCGATGCAACGCGAAAAACCTTACCTACCCTTGACATGTGCCGAAG
CCCGCTGAGAGGTGGGTGTGCCCGAAAGGGAGCGGCAACACAGG

Otu73 Core

GTAGTCCACCCCGTAAACGTTGGGAACTAGTTGTGGGGTCCATTCCACGGATTCCGTGACGCAGCTAACGCATTAAGTTCCCCGCCTGGGGAGTACGGCCGCA
AGGCTAAAACTCAAAGGAATTGACGGGGACCCGCACAAGCGGCGGAGCATGCGGATTAATTCGATGCAACGCGAAGAACCTTACCAAGGCTTGACATATACGA
GAACGGGCCAGAAATGGTCAACTCTTTGGACACTCGTAAACAGG

Otu84 Core

GTAGTCCACGCTGTAAACGATGAATGCTAGCCGTTGGGGTGCATGCACCTCAGTGGCGCCGCTAACGCATTAAGCATTCCGCCTGGGGAGTACGGTCGCAAGA
TTAAAACTCAAAGGAATTGACGGGGGCCCGCACAAGCGGTGGAGCATGTGGTTTAATTCGAAGCAACGCGCAGAACCTTACCATCCCTTGACATGGCATGCGA
GCCGGAGAGATCCGGTGTTCCCTTCGGGGACGTGCACACAGGTG

Otu87

GTAGTCCACGCCCTAAACGATGTCAACTGGTTGTTGGGTCTTCACTGACTCAGTAACGAAGCTAACGCGTGAAGTTGACCGCCTGGGGAGTACGGCCGCAAGG
TTGAAACTCAAAGGAATTGACGGGGACCCGCACAAGCGGTGGATGATGTGGTTTAATTCGATGCAACGCGAAAAACCTTACCCACCTTTGACATGTACGGAAT
CCTTTAGAGATAGAGGAGTGCTCGAAAGAGAGCCGTAACACAGG

Otu92 Core

GTAGTCCACGCTGTAAACGATGAATGCTAGCCGTTGGGGTGCATGCACCTCAGTGGCGCCGCTAACGCATTAAGCATTCCGCCTGGGGAGTACGGTCGCAAGA
TTAAAACTCAAAGGAATTGACGGGGGCCCGCACAAGCGGTGGAGCATGTGGTTTAATTCGAAGCAACGCGCAGAACCTTACCATCCCTTGACATGGCATGTTA
GCTCGAGAGATCGGGTGTCCTCTTCGGAGGCGTGCACACAGGTG

Otu94

GTAGTCCACGCCCTAAACGATGTCAACTAGCTGTTGGGGCCTTCGGGCCTTGGTAGCGCAGCTAACGCGTGAAGTTGACCGCCTGGGGAGTACGGTCGCAAGA
TTAAAACTCAAAGGAATTGACGGGGACCCGCACAAGCGGTGGATGATGTGGATTAATTCGATGCAACGCGAAAAACCTTACCTACCCTTGACATGTCTGGAAT
GCCGAAGAGATTTGGTAGTGCTCGCAAGAGAACCGGAACACAGG

Otu95

GTAGTCCACGCCGTAAACGATGGGTGCTAGACGTTGGCGAGCTTGCTCGTCAGTGTCGCAGCTAACGCGTTAAGCACCCCGCCTGGGGAGTACGGCCGCAAGG
TTGAAACTCAAAGGAATTGACGGGGGCCCGCACAAGCGGTGGAGCATGTGGTTCAATTCGACGCAACGCGCAGAACCTTACCAGCCCTTGACATGGGACTCGC
CGGGAGCAGAGACGCTCCCTTCGGTTCGGCCGGAGTCCGCACAG

Otu98 Core

GTAGTCCACGCCGTAAACGATGAAGACTAGCTGTCCGGGCACATGGTGCTTGGGTGGCGCAGCTAACGCATTAAGTCTTCCGCCTGGGGAGTACGGCCGCAAG
GTTAAAACTCAAAGAAATTGACGGGGGCCTGCACAAGCGGTGGAGCATGTGGTTTAATTCGAAGCAACGCGCAGAACCTTACCAGCGTTTGACATGTCCGGAC
GATTTCCAGAGATGGATCTCTTCCCTTCGGGGACTGGAACACAG

Otu99 Core

GTAGTCCACGCCGTAAACGATGAATGTTAGCCGTCGGGCAGTATACTGTTCGGTGGCGCAGCTAACGCATTAAACATTCCGCCTGGGGAGTACGGTCGCAAGA
TTAAAACTCAAAGGAATTGACGGGGGCCCGCACAAGCGGTGGAGCATGTGGTTTAATTCGAAGCAACGCGCAGAACCTTACCAGCCCTTGACATCCTACGACC
GCTGCAGAGATGCAGTTTCCACTTCGGTGGCGTAGAGACAGGTG

Otu104 Core

GTAGTCCACGCCGTAAACGATGAATGCCAGCTGTTGGGGTGCTTGCACCGCAGTAGCGCAGCTAACGCTTTGAGCATTCCGCCTGGGGAGTACGGTCGCAAGA
TTAAAACTCAAAGGAATTGACGGGGGCCCGCACAAGCGGTGGAGCATGTGGTTTAATTCGAAGCAACGCGCAGAACCTTACCATCCTTTGACATGGCGTGTTA
CCCAGAGAGATCTGGGGTCCCCTTCGGGGGCGCGCACACAGGTG

Otu107

GTAGTCCACGCCCTAAACGATGTCTACTAGTTGTTGGTGGAGTAAAATCCATGAGTAACGCAGCTAACGCGTGAAGTAGACCGCCTGGGGAGTACGGTCGCAA
GATTAAAACTCAAAGGAATTGACGGGGGCCCGCACAAGCGGTGGATTATGTGGATTAATTCGATGCAACGCGAAAAACCTTACCTGGCCTTGACATGCCACTA
ACGAAGCAGAGATGCATTAGGTGCCCGTAAGGGAAAGTGGACAC

Otu108 Core

GTAGTCCACGCCCTAAACGATGTCGACTGGGTGTTGGGGAAGCGATTTCCCAGTACCGTAGCTAACGCGTGAAGTCGACCGCCTGGGGAGTACGGCCGCAAGG
TTAAAACTCAAAGGAATTGACGGGGACCCGCACAAGCGGTGGATGATGTGGATTAATTCGATGCAACGCGAACAACCTTACCTACCCTTGACATGTCCGAAAG
CCCGCTGAGAGGCGGGGGTGCCCGAAAGGGAATCGGAACACAGG

Otu112

GTAGTCCACGCCGTAAACTATGGGTGCTAGCCGTCGGTCGGCATGCCGGTCGGTGGCGCAGCAAACGCATTAAGCACCCCGCCTGGGGAGTACGGTCGCAAGA
TTAAAACTCAAAGGAATTGACGGGGGCCCGCACAAGCGGTGGAGCATGTGGTTTAATTCGAGGCAACGCGAAGAACCTTACCAGCCCTTGACTTCCCGATCGG
GGCTCCGGAGACGGAGCTCTTCAATTGGTCGGTGACAGGTGCTG

Otu116

GTAGTCCACGCCGTAAACTATGAGAGCTAGCCGTTGGAGAGTTTACTCTTCAGTGGCGCAGCTAACGCATTAAGCTCTCCGCCTGGGGAGTACGGTCGCAAGA
TTAAAACTCAAAGGAATTGACGGGGGCCCGCACAAGCGGTGGAGCATGTGGTTTAATTCGAAGCAACGCGAAGAACCTTACCAGCCCTTGACATGGCAGGACG
GTTTCCAGAGATGGATTCCTTCACTTCGGTGACTTGCACACAGG

Otu127	GTAGTCCACGCCCTAAACGATGCGAACTGGATGTTGGGTGCAACTTGGCACCCAGTATCGAAGCTAACGCGTTAAGTTCGCCGCCTGGGGAGTACGGTCGCAA<br>GACTGAAACTCAAAGGAATTGACGGGGGCCCGCACAAGCGGTGGAGTATGTGGTTTAATTCGATGCAACGCGAAGAACCTTACCTGGTCTTGACATCCACGGA<br>ACTTTCCAGAGATGGATTGGTGCCTTCGGGAACCGTGAGACAGG
**Otu131 Core**	GTAGTCCACGCCGTAAACGATGGATGCTAGCCGTTGGCAGGCTTGCCTGTCAGTGGCGCAGCTAACGCATTAAGCATCCCGCCTGGGGAGTACGGTCGCAAGA<br>TTAAAACTCAAAGGAATTGACGGGGGCCCGCACAAGCGGTGGAGCATGTGGTTCAATTCGAAGCAACGCGCAGAACCTTACCAACCTTTGACATGTCCCGTAT<br>GGGCACCAGAGATGGAGCCCTTCAGTTCGGCTGGCGGGAACACA
**Otu169 Core**	GTAGTCCACGCCGTAAACGATGATGACTAGCTGTCGGGGCTCATGGAGTTTCGGTGGCGCAGCTAACGCGTTAAGTCATCCGCCTGGGGAGTACGGCCGCAAG<br>GTTAAAACTCAAAGAAATTGACGGGGGCCTGCACAAGCGGTGGAGCATGTGGTTTAATTCGAAGCAACGCGCAGAACCTTACCAGCGTTTGACATGGTAGGAC<br>GGTTTCCAGAGATGGATTCCTTCCCTTACGGGACCTACACACAG
**Otu170 Core**	GTAGTCCACGCCCTAAACGATGTCTACTAGTTGTCGGGTCTTAATTGACTTGGTAACGCAGCTAACGCGTGAAGTAGACCGCCTGGGGAGTACGGTCGCAAGA<br>TTAAAACTCAAAGGAATTGACGGGGACCCGCACAAGCGGTGGATGATGTGGATTAATTCGATGCAACGCGAAAAACCTTACCTACCCTTGACATGGAAGGAAT<br>CCTTGAGAGATTGAGGAGTGCCCGAAAGGGAACCTTTACACAGG
**Otu403 Core**	GTAGTCCACGCCGTAAACGATGATAACTAGCTGTCCGGTCACTTGGTGATTGGGTGGCGCAGCTAACGCATTAAGTTATCCGCCTGGGGAGTACGGCCGCAAG<br>GTTAAAACTCAAAGGAATTGACGGGGGCCTGCACAAGCGGTGGAGCATGTGGTTTAATTCGAAGCAACGCGCAGAACCTTACCAGCGTTTGACATGGTAGGAC<br>GACTGGCAGAGATGCCTTTCTTCCCTTCGGGGACCTACACACAG
**Otu1132 Core**	GTAGTCCACGCTGTAAACGATGAATGCTAGCCGTAGGGGTGCATGCACTTCTGTGGCGCCGCTAACGCATTAAGCATTCCGCCTGGGGAGTACGGTCGCAAGA<br>TTAAAACTCAAAGGAATTGACGGGGGCCCGCACAAGCGGTGGAGCATGTGGTTTAATTCGAAGCAACGCGCAGAACCTTACCATCCCTTGACATGGCATGTTA<br>CATGGAGAGATCCATGGTCCTCTTCGGAGGCGTGCACACAGGTG

Table S12 Examples from the literature where core and candidate-core taxa identified in this study have been reported to be important to *Musa* spp. plant fitness or associated with the plant.

OTUs	Taxonomy	Culture independent Study?	Comparison/ management intervention	Outcome	Link to plant fitness	References
17, 35, 3	*Bacillus*	Both	Inoculation of *Bacillus*	Increase in relative abundance	Negatively associated with Fusarium Wilt and Banana Bunchy Top Virus symptoms	[3, 11, 12, 19, 21, 23-28]
		Yes	Fusarium Wilt affected plants vs. healthy	Increases in abundance or shifts between different *Bacillus* species	Negatively associated with Fusarium Wilt symptoms	[21,29,30]
		No	Co-culture with *Fusarium oxysporum* f. sp. *cubense*	-	Suppression of plant pathogen on plates	[18]
			None applied – reported to be associated with *Musa* spp.	No change		[9, 13, 16, 17]
9	*Bradyrhizobium*	Yes	Pineapple plant rotation with *Musa* spp.	Increased relative abundance	Negatively associated with Fusarium Wilt symptoms	[20]
			Fusarium Wilt affected plants vs. healthy	Increased relative abundance	Highest in Fusarium Wilt effected plants or near to effected plants but not showing symptoms	[29]
			Fusarium Wilt affected plants vs. healthy	Increased relative abundance	Positively associated with Fusarium Wilt symptoms	[31]
			Fusarium Wilt affected plants vs. healthy	No change		[30]

61	*Curtobacterium*	No	None applied – reported to be associated with *Musa* spp.	No change	-	[18]
116	*Devosia*	Yes	Lime applied to soil	Increased relative abundance	Lower plant biomass in first 65 days of growth	[32]
			Fusarium Wilt affected plants vs. healthy and examination of tissue culture plantlets	No change	Present in all adult plants regardless of Wilt but not found in tissue culture plants	[29]
			Addition of organic matter to soil in which plant is grown	Increased relative abundance	Negatively associated with Fusarium Wilt symptoms	[31]
			Bacterial Wilt affected plants vs. healthy	Not present during wilting	-	[10]
20	*Dyella*	Yes	Bacterial Wilt affected plants vs. healthy	Not present during wilting	-	[14]
8	*Enterobacteria*	Yes	Location, agroforestry co-production, herbicide use, Fusarium Wilt affected plants vs. healthy, and Bacterial Wilt affected plants vs. healthy	No change	-	[7,8,14,30]
		No	None applied – reported to be associated with *Musa* spp.	No change	-	[9,13,15,18,33]
40	*Gaiella*	Yes	Fusarium Wilt affected plants vs. healthy or Bacterial Wilt affected plants vs. healthy	Decrease in relative abundance	Negatively associated with Fusarium Wilt symptoms	[26]
			Soil fumigation before growing plants affected by Fusarium Wilt	Fumigation increased abundance	Negatively associated with Fusarium Wilt symptoms	[28]

23	*Gemmatimonadaceae*	Yes	Fusarium Wilt affected plants vs. healthy, *Bacillus* inoculated compost, or chili rotation	Increase when treatment results in Fusarium Wilt decrease	Positively associated with an increase in plant biomass	[11,27,31,34]
			Addition of *Bacillus* inoculated sterile compost to control Fusarium Wilt	Decreased relative abundance	Positively associated with Fusarium Wilt symptoms	[21]
			Plants grown in fields with a different history of monoculture. Plants are either affected by Fusarium Wilt or healthy	Dominant regardless of treatment	-	[26]
			Bacterial Wilt affected plants vs. healthy	Not present during wilting	-	[14]
92, 104, 1132, 84	*Methylobacterium-Methylorubum*	Yes	Fusarium Wilt affected plants vs. healthy or Bacterial Wilt affected plants vs. healthy	Present regardless	-	[29,14]
		No	None applied – reported to be associated with *Musa* spp.	No change	-	[35]
73	*Microbacterium*	Yes	Fusarium Wilt affected plants vs. healthy	Present in healthy plants near to those wilting and wilting plants	-	[29]
			Bacterial Wilt affected plants vs. healthy	No change	-	[14]
		Both	None applied – reported to be associated with *Musa* spp.	No change	-	[22,36]
16	*Mycobacterium*	Yes	Fusarium Wilt affected plants vs. healthy	Present in wilting plants only	Positively associated with Fusarium Wilt symptoms	[29]

			Fumigation and addition of *Bacillus* inoculated sterile compost or conventional compost	No change	-	[14]
			None applied – reported to be associated with *Musa* spp.	No change	-	[2,22]
28	*Nitrospira*	Yes	Addition of *Bacillus* inoculated compost to control Fusarium Wilt	No change	-	[11]
			Comparison of wilt suppressive and conducive fields	No change	-	[24]
			Bacterial Wilt affected plants vs. healthy	Not present under wilting conditions	-	[14]
			Soil fumigation to control Fusarium Wilt	Reduced	Negatively associated with Fusarium Wilt symptoms	[28]
			Plants grown in fields with a different history of monoculture. Plants are either affected by Fusarium Wilt or healthy	No change	-	[19]
42	*Pseudomonas*	No	Inoculation of *Pseudomonas* strains	Increase in relative abundance	Negatively associated with Banana Bunchy Top Virus symptoms	[23]
			None applied – reported to be associated with *Musa* spp.	No change	-	[15,5]
			Co-culture with *Fusarium oxysporum* f. sp. *cubense*	No change	Suppression of plant pathogen on plates	[18]
		Yes	Agroforestry co-production with *Musa* spp.	Increased relative abundance	-	[7]

Plants grown in different locations	Increased relative abundance	-	[8]
Fusarium Wilt affected plants vs. healthy	Changes in the composition of *Pseudomonas* spp.	-	[30]
Fusarium Wilt affected plants vs. healthy	Present regardless	-	[29]
Comparison of Fusarium Wilt suppressive and conducive fields	Increased relative abundance in suppressive field	Negatively associated with Fusarium Wilt symptoms	[24]
Bacterial Wilt affected plants vs. healthy	No change	-	[14]
Addition of *Bacillus* inoculated compost to control Fusarium Wilt	Increased relative abundance	Negatively associated with Fusarium Wilt symptoms	[21]
	No change	-	[25]
Addition of *Bacillus* inoculated compost after soil fumigation to control Fusarium Wilt	Decrease in relative abundance	Positively associated with Fusarium Wilt symptoms	[23]
Addition of *Bacillus* inoculated compost after soil fumigation to control Fusarium Wilt	Increase in relative abundance	Negatively associated with Fusarium Wilt symptoms	[27]
Addition of pig manure	Increase in relative abundance		[28]
Compared with a chili plant rotation over time	Found in only one of two years recorded but negatively associated with *Fusarium oxysporum* f. sp. *cubense*	Negatively associated with Fusarium Wilt symptoms	[34]

			Agroforestry co-production with banana or herbicide used	Clustering of *Pseudomonas* communities depending on treatment	-	[10]
10	*Ralstonia*	Yes	Fusarium Wilt affected plants vs. healthy	Decrease in relative abundance	Negatively associated with Fusarium Wilt symptoms	[30]
			Fusarium Wilt affected plants vs. healthy	Present in wilting plants and those not showing symptoms next to wilting	-	[29]
			Addition of *Bacillus* inoculated compost to control Fusarium Wilt	Decrease in relative abundance	Positively associated with Fusarium Wilt symptoms	[36]
			Bacterial Wilt affected plants vs. healthy	No change	-	[14]
2, 99, 11	*(Allo,Neo,Para) Rhizobium*	No	None applied – reported to be associated with *Musa* spp.	No change	-	[13,9,5]
			Inoculation	Increased abundance	Increased plant biomass	[33]
		Yes	Bacterial Wilt affected plants vs. healthy	No change	-	[14]
			Fusarium Wilt affected plants vs. healthy	Found in wilting plants and those that were healthy next to wilting plants	-	[29]
			Addition of *Bacillus* inoculated compost to control Fusarium Wilt	Highest under conventional fertiliser (most abundant genus)	Positively associated with Fusarium Wilt symptoms	[31]
			Addition of *Bacillus* inoculated compost and fumigation to control Fusarium Wilt	No change	-	[36]

			Addition of *Bacillus* inoculated compost and fumigation to control Fusarium Wilt	No change	-	[28]
21	*Sphingobium*	Yes	Agroforestry co-production with banana or herbicide used	No change	-	[10]
			Compared with a chili plant rotation over time	Found in only one of two years recorded but negatively associated with *Fusarium oxysporum* f. sp. *cubense*	Negatively associated with Fusarium Wilt symptoms	[34]
			Addition of *Bacillus* inoculated compost to control Fusarium Wilt vs. healthy	Increased abundance	Negatively associated with Fusarium Wilt symptoms	[21,36]
				No change	-	[31]
			Fusarium Wilt affected plants vs. healthy	Found in wilting plants and those that were healthy next to wilting plants	-	[29]
			Bacterial Wilt affected plants vs. healthy	No change	-	[14]
98, 12, 169, 403, 4	*Sphingomonas*	No	None applied – reported to be associated with *Musa* spp.	No change	-	[18]
		Yes	Addition of *Bacillus* inoculated compost to control Fusarium Wilt	Increased abundance	Negatively associated with Fusarium Wilt symptoms	[11,21]
			Addition of *Bacillus* inoculated compost to control Fusarium Wilt	Increased under low addition, high addition no different from control	Negatively associated with Fusarium Wilt symptoms	[25]

			Changes in fertiliser management	Highest under conventional fertiliser	Positively associated with Fusarium Wilt symptoms	[31]
			Plants grown in fields with a different history of monoculture. Plants are either affected by Fusarium Wilt or healthy	Lower abundance	Negatively associated with Fusarium Wilt symptoms and length of time in monoculture	[26]
			Addition of *Bacillus* inoculated compost and fumigation to control Fusarium Wilt	Increased abundance	Negatively associated with Fusarium Wilt symptoms	[28]
			Lime applied to soil	Increased abundance	Lower plant biomass in first 65 days of growth	[32]
			Compared with a chili plant rotation over time	Found in only one of two years recorded but negatively associated with *Fusarium oxysporum* f. sp. *cubense*	Negatively associated with Fusarium Wilt symptoms	[34]
			Fusarium Wilt affected plants vs. healthy	Found in tissue culture plants, wilting plants and those that were healthy next to wilting plants	-	[29]
25	*Streptomyces*	Both	None applied – reported to be associated with *Musa* spp.	No change	-	[17]
		Yes	Addition of organic matter to soil	Increased abundance	-	[32]
			Fusarium Wilt affected plants vs. healthy	Found in wilting plants and those that were healthy next to wilting plants	-	[29]

			Addition of *Bacillus* inoculated compost to control Fusarium Wilt	Increased abundance	Negatively associated with Fusarium Wilt symptoms	[21]
			Addition of *Bacillus* inoculated compost and fumigation to control Fusarium Wilt	No change	-	[28]
		Both	Inoculation of *Streptomyces*	Increase in relative abundance	Negatively associated with Fusarium Wilt symptoms	[2]

References

1. De la Torre-González FJ, Fernández-Castillo E, Azaharez-Llorente D, Lara J, Avendaño E, Castañeda A, et al. Response to Edaphoclimatic Conditions and Crop Management of the Bacterial Microbiome of Musa acuminata Rhizosphere Profiled by 16S rRNA Gene Amplicon Sequencing. Microbiol Resour Announc. 2021;10:1–4.
2. Du X, Zhai Y, Deng Q, Tan H, Cao L. Illumina-Based Sequencing Analysis Directed Selection for Actinobacterial Probiotic Candidates for Banana Plants. Probiotics Antimicrob Proteins. 2018;10:284–92.
3. Fu L, Ruan Y, Tao C, Li R, Shen Q. Continuous application of bioorganic fertilizer induced resilient culturable bacteria community associated with banana Fusarium wilt suppression. Sci Rep. 2016;6:27731.
4. Gómez-Lama Cabanás C, Fernández-González AJ, Cardoni M, Valverde-Corredor A, López-Cepero J, Fernández-López M, et al. The Banana Root Endophytome: Differences between Mother Plants and Suckers and Evaluation of Selected Bacteria to Control Fusarium oxysporum f.sp. cubense. J Fungi. 2021;7:194.
5. Karthik M, Pushpakanth P, Krishnamoorthy R, Senthilkumar M. Endophytic bacteria associated with banana cultivars and their inoculation effect on plant growth. J Hortic Sci Biotechnol. 2017;92:568–76.
6. Kaushal M, Swennen R, Mahuku G. Unlocking the microbiome communities of banana (Musa spp.) under disease stressed (fusarium wilt) and non-stressed conditions. Microorganisms. 2020;8:1–19.
7. Köberl M, Dita M, Martinuz A, Staver C, Berg G. Agroforestry leads to shifts within the gammaproteobacterial microbiome of banana plants cultivated in Central America. Front Microbiol. 2015;6:1–10.

8. Köberl M, Dita M, Martinuz A, Staver C, Berg G. Members of Gammaproteobacteria as indicator species of healthy banana plants on Fusarium wilt-infested fields in Central America. Sci Rep. 2017;7:45318.
9. Pereira DFG., Nietsche S, Xavier AA, de Souza SA, Costa MR, Duarte AB. Characterization and activity of endophytic bacteria from 'Prata Anã' banana crop (Musa sp., AAB). Revista Ceres. 2018;65:381–7
10. Rossmann B, Müller H, Smalla K, Mpiira S, Tumuhairwe JB, Staver C, et al. Banana-associated microbial communities in Uganda are highly diverse but dominated by Enterobacteriaceae. Appl Environ Microbiol. 2012;78:4933–41.
11. Shen Z, Wang D, Ruan Y, Xue C, Zhang J, Li R, et al. Deep 16S rRNA pyrosequencing reveals a bacterial community associated with banana Fusarium wilt disease suppression induced by bio-organic fertilizer application. PLoS One. 2014;9:1–10.
12. Shen Z, Wang B, Lv N, Sun Y, Jiang X, Li R, et al. Effect of the combination of bio-organic fertiliser with *Bacillus* amyloliquefaciens NJN-6 on the control of banana Fusarium wilt disease, crop production and banana rhizosphere culturable microflora. Biocontrol Sci Technol. 2015;25:716–31.
13. Souza SA, Xavier AA, Costa MR, Cardoso AMS, Pereira MCT, Nietsche S. Endophytic bacterial diversity in banana "Prata Anã" (Musa spp.) roots. Genet Mol Biol. 2013;36:252–64..
14. Suhaimi NSM, Goh SY, Ajam N, Othman RY, Chan KG, Thong KL. Diversity of microbiota associated with symptomatic and non-symptomatic bacterial wilt-diseased banana plants determined using 16S rRNA metagenome sequencing. World J Microbiol Biotechnol. 2017;33:1–10.

15. Thomas P, Swarna GK, Roy PK, Patil P. Identification of culturable and originally non-culturable endophytic bacteria isolated from shoot tip cultures of banana cv. Grand Naine. Plant Cell Tissue Organ Cult. 2008;93:55–63.

16. Thomas P, Soly TA. Endophytic Bacteria Associated with Growing Shoot Tips of Banana (Musa sp.) cv. Grand Naine and the Affinity of Endophytes to the Host. Microb Ecol. 2009;58:952–64.

17. Thomas P, Sekhar AC. Cultivation Versus Molecular Analysis of Banana (Musa sp.) Shoot-Tip Tissue Reveals Enormous Diversity of Normally Uncultivable Endophytic Bacteria. Microb Ecol. 2017;73:885–99.

18. Sekhar AC, Thomas P. Isolation and Identification of Shoot - Tip Associated Endophytic Bacteria from Banana cv. Grand Naine and Testing for Antagonistic Activity against Fusarium oxysporum f. sp. cubense. Am J Plant Sci. 2015;6:943–54.

19. Wang B, Yuan J, Zhang J, Shen Z, Zhang M, Li R, et al. Effects of novel bioorganic fertilizer produced by *Bacillus* amyloliquefaciens W19 on antagonism of Fusarium wilt of banana. Biol Fert Soils. 2013;49:435–46.

20. Wang B, Li R, Ruan Y, Ou Y, Zhao Y, Shen Q. Pineapple-banana rotation reduced the amount of Fusarium oxysporum more than maize-banana rotation mainly through modulating fungal communities. Soil Biol Biochem. 2015;86:77–86.

21. Xue C, Penton CR, Shen Z, Zhang R, Huang Q, Li R, et al. Manipulating the banana rhizosphere microbiome for biological control of Panama disease. Sci Rep. 2015;5:11124.

22. Zhai Y, Wang W, Tan H, Cao L. A New Approach to Analyzing Endophytic Actinobacterial Population in the Roots of Banana Plants (Musa sp., AAA). J Biochem Mol Biol Res. 2016;2:180–4.

23. Harish S, Kavino M, Kumar N, Saravanakumar D, Soorianathasundaram K, Samiyappan R. Biohardening with Plant Growth Promoting Rhizosphere and Endophytic bacteria

induces systemic resistance against Banana bunchy top virus. Appl Soil Ecol. 2008;39:187–200.

24. Shen Z, Ruan Y, Xue C, Zhong S, Li R, Shen Q. Soils naturally suppressive to banana Fusarium wilt disease harbor unique bacterial communities. Plant Soil. 2015;393:21–33.
25. Shen Z, Ruan Y, Wang B, Zhong S, Su L, Li R, et al. Effect of biofertilizer for suppressing Fusarium wilt disease of banana as well as enhancing microbial and chemical properties of soil under greenhouse trial. Appl Soil Ecol. 2015;93:111–9.
26. Shen Z, Penton CR, Lv N, Xue C, Yuan X, Ruan Y, et al. Banana fusarium wilt disease incidence is influenced by shifts of soil microbial communities under different monoculture spans. Microb Ecol. 2018;75:739–50.
27. Shen Z, Xue C, Penton CR, Thomashow LS, Zhang N, Wang B, et al. Suppression of banana Panama disease induced by soil microbiome reconstruction through an integrated agricultural strategy. Soil Biol Biochem. 2019;128:164–74.
28. Zhang N, He X, Zhang J, Raza W Yang XM, Ruan YZ, et al. Suppression of Fusarium Wilt of Banana with Application of Bio-Organic Fertilizers. Pedosphere. 2014;24:613–24.
29. Liu Y, Zhu A, Tan H, Cao L, Zhang R. Engineering banana endosphere microbiome to improve Fusarium wilt resistance in banana. Microbiome. 2019;7:1–15.
30. Lian J, Wang ZF, Zhou SN. Response of endophytic bacterial communities in banana tissue culture plantlets to Fusarium wilt pathogen infection. J Gen Appl Microbiol. 2008;54:83–92.
31. Shen Z, Ruan Y, Chao X, Zhang J, Li R, Shen Q. Rhizosphere microbial community manipulated by 2 years of consecutive biofertilizer application associated with banana Fusarium wilt disease suppression. Biol Fertil Soils. 2015;51:553–62.

32. Zhang J, Bei S, Li B, Zhang J, Christie P, Li X. Organic fertilizer, but not heavy liming, enhances banana biomass, increases soil organic carbon and modifies soil microbiota. Appl Soil Ecol. 2019;136:67–79.

33. Martínez L, Caballero-Mellado J, Orozco J, Martínez-Romero E. Diazotrophic bacteria associated with banana (Musa spp.). Plant Soil. 2003;257:35–47.

34. Hong S, Jv H, Lu M, Wang B, Zhao Y, Ruan Y. Significant decline in banana Fusarium wilt disease is associated with soil microbiome reconstruction under chilli pepper-banana rotation. Eur J Soil Biol. 2020;97:103154.

35. Thomas P, Swarna GK, Patil P, Rawal RD. Ubiquitous presence of normally non-culturable endophytic bacteria in field shoot-tips of banana and their gradual activation to quiescent cultivable form in tissue cultures. Plant Cell Tissue Organ Cult. 2008;93:39–54.

36. Fu L, Penton CR, Ruan Y, Shen Z, Xue C, Li R, et al. Inducing the rhizosphere microbiome by biofertilizer application to suppress banana Fusarium wilt disease. Soil Biol Biochem. 2017;104:39–48.
